# Most myopathic lamin variants aggregate: a functional genomics approach for assessing variants of uncertain significance

**DOI:** 10.1038/s41525-021-00265-x

**Published:** 2021-12-03

**Authors:** Corey L. Anderson, Emma R. Langer, Timothy C. Routes, Seamus F. McWilliams, Igor Bereslavskyy, Timothy J. Kamp, Lee L. Eckhardt

**Affiliations:** 1 Department of Medicine, University of Wisconsin-Madison, Madison, WI 53705 USA; 2 Cellular and Molecular Arrhythmias Research Program, University of Wisconsin-Madison, Madison, WI 53705 USA; 3 Department of Cell and Regenerative Biology, University of Wisconsin-Madison, Madison, WI 53705 USA

**Keywords:** Functional genomics, Medical genetics

## Abstract

Hundreds of *LMNA* variants have been associated with several distinct disease phenotypes. However, genotype–phenotype relationships remain largely undefined and the impact for most variants remains unknown. We performed a functional analysis for 178 variants across five structural domains using two different overexpression models. We found that lamin A aggregation is a major determinant for skeletal and cardiac laminopathies. An in vitro solubility assay shows that aggregation-prone variants in the immunoglobulin-like domain correlate with domain destabilization. Finally, we demonstrate that myopathic-associated *LMNA* variants show aggregation patterns in induced pluripotent stem cell derived-cardiomyocytes (iPSC-CMs) in contrast to non-myopathic *LMNA* variants. Our data-driven approach (1) reveals that striated muscle laminopathies are predominantly protein misfolding diseases, (2) demonstrates an iPSC-CM experimental platform for characterizing laminopathic variants in human cardiomyocytes, and (3) supports a functional assay to aid in assessing pathogenicity for myopathic variants of uncertain significance.

## Introduction

The nuclear lamina is composed of the intermediate filament protein lamins A/C, B1 and B2 encoded by *LMNA*, *LMNB1*, and *LMNB2*, respectively^1^. Through interactions with numerous integral membrane proteins, the nuclear lamina performs myriad functions such as providing shape and support to the nucleus, linking the nucleus with the cytoplasm, regulating transcription, and serving as a platform for proteins involved in signal transduction^1^. Over 600 missense *LMNA* variants (the dominant variant type) have been reported in ClinVar, a variant interpretation database, and are associated with more clinical disease phenotypes than any other gene, collectively referred to as laminopathies^2^. Each can largely be grouped into one or more of four broad categories with ~80% associated with autosomal dominant (a) skeletal muscle (e.g., Emery-Dreifuss Muscular Dystrophy)^3^ or (b) cardiac muscle disease (e.g., Dilated Cardiomyopathy (DCM))^4^ with the remaining <20% associated with (c) lipodystrophy (e.g., Dunnigan type familial partial lipodystrophy)^5^ or the extremely rare (d) premature aging syndromes (e.g., Hutchinson-Gilford progeria syndrome (HGPS))^6^. In addition, a homozygous recessive Charcot^_^Marie-Tooth Disease (CMT2B1)—associated variant has also been characterized^7^.

Lamins have a short N-terminal domain, long rod domain consisting of four coiled-coil domains (CCDs) (1A, 1B, 2A, and 2B)^8^, and a C-terminal protein interaction rich immunoglobulin-like domain (IgD)^9^, which polymerize to form intermediate filaments in the nucleus. For skeletal and heart muscle cells, where lamin A/C is highly expressed, mechanical stress and altered gene expression hypotheses have been suggested, and both mechanisms likely contribute to cardiac and skeletal myopathies^10,11^. For lipodystrophy, mechanisms of altered adipocyte differentiation and extracellular matrix have been described among others^12^. For premature aging syndromes, the best-understood mechanism is abnormal prelamin A processing due to a de novo cryptic splice site variant (*LMNA* c.1824C > T, p.G608G) resulting in a 50aa deletion protein called progerin^13^ that causes nuclear deformation. In fact, nuclear membrane abnormalities including “blebs”, “honeycombs”, and lamin A/C foci are a hallmark of all classes of laminopathies^14–16^.

Despite these mechanisms, ascribing *LMNA* variants to tissue-specific phenotypes is a challenge. Further, most *LMNA* variants have not been functionally characterized with over 400 remaining classified as variants of uncertain significance (VUS) in ClinVar. To address the challenges of genotype–phenotype relationships and pathogenicity, we undertook a systematic lamin A analysis of 178 missense variants - the most common variant type—across five structural domains using two mammalian overexpression models (HEK 293 cells and mouse C2C12 myoblasts). We studied aggregation because (1) misfolding and aggregation are dominant mechanisms underlying inherited diseases and may help reveal genotype–phenotype relationships^17^, (2) aggregation of several *LMNA* variants has been reported in a variety of model systems^10,14,15,18–20^, and (3) it could serve as a relatively simple functional test to support pathogenic classification as defined by the American College of Medical Genetics (ACMG) guidelines for variant classification^21^. We then tested IgD stability for each variant to compare with the lamin A aggregation results^22^. Finally, we evaluated the pathogenicity and stability prediction tools FoldX^23^ and REVEL^24^ for each LMNA variant characterized. In this study, we show that up to 78% of striated muscle disease (84% skeletal and 52% cardiac) variants tested form aggregates in the nucleus, whereas only 35% of lipodystrophy and 17% of progeria variants do; the latter two only showing aggregation when a skeletal muscle phenotype is also present. Overall, REVEL performed well for myopathic variants but not for other types. In addition, aggregation for variants in the IgD is associated with loss of domain stability and misfolding and supported by FoldX analysis with only a few exceptions. Combined, our results could serve as a functional test as part of supporting data toward pathogenic classification as defined by the ACMG^21^. Finally, we show lamin A aggregation in human induced pluripotent stem cell derived-cardiomyocytes (iPSC-CMs) for a subset of variants, further validating aggregation as a major determinant of striated muscle laminopathies. These results (1) are a valuable resource for further studies investigating laminopathies, (2) demonstrate an in vitro functional assay to assist in classifying *LMNA* VUS associated with striated muscle disease, (3) establish a human cardiac cell model for studying cardiac laminopathies, and (4) point to lamin A aggregation as a potential therapeutic target for the majority of myopathic variants.

## Results

### Selection of *LMNA* variants for functional studies

**Fig. 1:**
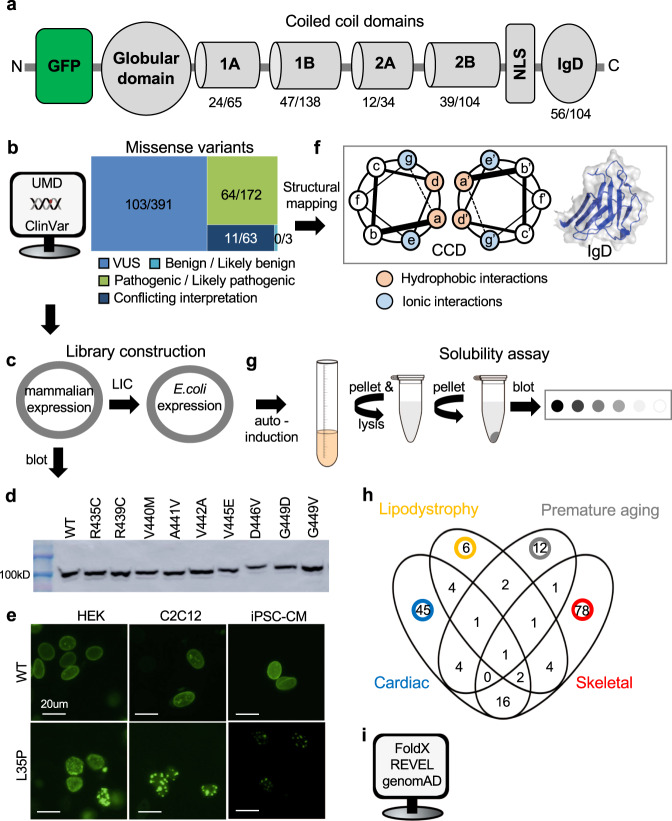
Lamin characterization workflow. **a** Schematic of lamin A showing each structural domain and locations of the GFP tag and NLS. Numbers indicate the number of missense variants from Universal Mutational Database studied herein/total number of missense variants in ClinVar (and UMD) for indicated domains. **b** Sequence variants grouped by their clinical significance are described in ClinVar (linker variants not included). Numbers for each category indicate the number of missense variants we studied/total number of missense variants reported in ClinVar as of July 2021. **c** Variant library construction. **d** Representative western blots of full-length lamin A expressed in HEK 293 cells (lamin A (74kD) + GFP (27kD) = 101kD). **e** Representative images (20 μm scale bar) of L35P nuclear aggregation compared to WT for the three cell types used. **f** Mapping of the variants to the IgD structure and CCD locations (pink and blue indicate hydrophobic and ionic dimerization interacting residues, respectively and gray indicates variable surface residues. **g** Solubility (i.e., stability) assay described previously^22^. **h** Venn diagram showing disease classification for all disease-linked variants studied. **i** In silico tools used to compare to lamin A variant aggregation results. *GFP* green fluorescent protein, *NLS* nuclear localization signal, IgD immunoglobin-like domain, UMD, Universal Mutational Database, *VUS* variants of uncertain significance, *CCD* coiled-coil domain, *LIC* ligation-independent subcloning, *HEK* human embryonic kidney cells, mouse myoblasts (C2C12), *iPSC-CMs* induced pluripotent stem cell-induced cardiomyocytes, *REVEL* Rare Exome Ensemble Learner, *genomAD* Genome Aggregation Database.

We chose to perform a comprehensive analysis of all *LMNA* missense variants listed in the Universal Mutational Database (UMD-LMNA) in lamin A’s five structural domains (no linker regions) and several in the much larger ClinVar database that occur in the general population, which are included as negative controls (Fig. 1a, Supplementary Tables 1–5). We chose UMD missense variants because they list associated phenotypes for all variants to help draw genotype–phenotype relationships. We chose to study missense variants in structural regions to gain insight into structure-(dys)function relationships toward more definitive variant classification. Next, based on ClinVar, all 178 missense variants were classified as either pathogenic/likely pathogenic (64), VUS (103), or conflicting interpretation (11). None were reported as benign/likely benign. (Fig. 1b, Supplementary Tables 1–5). We then generated a cDNA library containing all 178 variants in the CCDs and IgD in a mammalian expression vector and 56 IgD variants in an *Escherichia coli* expression vector for functional studies (Fig. 1c). At least one western blot was performed to confirm the correct size for each variant in HEK 293 cells with several representative examples shown in Fig. 1d and Supplementary Figure 1. A comprehensive image-based lamin A aggregation analysis was performed for all variants in both HEK 293 cells and C2C12 myoblasts and for a small subset iPSC-CMs. Figure 1e shows representative images of diffuse GFP-WT lamin A nuclear expression in contrast to nuclear foci or aggregates using GFP-L35P lamin A as an example (see Supplementary Figures 2 and 3 for more examples). Each variant was also mapped to helical locations of the CCD^8^ and IgD crystal structure^9^ to identify any potential structure-phenotype correlations (Fig. 1f). A solubility assay we recently reported was then used as a proxy to test each variant’s effects on IgD stability (Fig. 1g)^22^. All variants that we characterized by aggregation were then grouped into one of four clinical phenotypic categories (104 skeletal muscle disease (red), 73 cardiac muscle disease (blue), 21 lipodystrophy (yellow), or 20 premature agings (gray)) in order to identify potential genotype–phenotype correlations (Fig. 1h). Some variants were associated with more than one clinical phenotype with the most frequent overlap being between cardiomyopathy and skeletal muscular dystrophy. Finally, we tested two in silico pathogenic prediction tools FOLDX^23^ and REVEL^24^ against our large aggregation data set (Fig. 1i).

### Aggregation propensity of *LMNA* variants in the CCDs

**Fig. 2:**
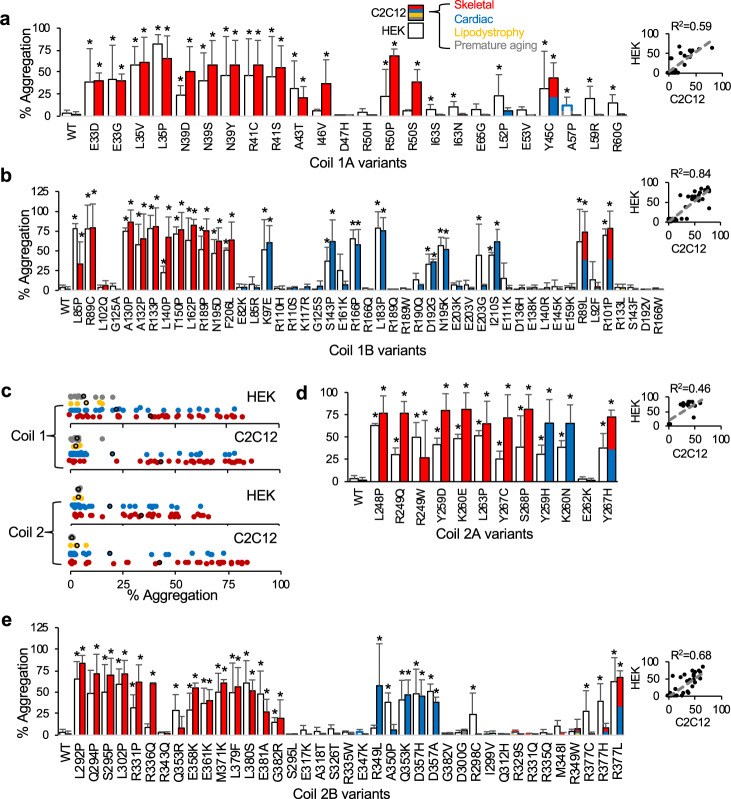
Lamin A aggregation for CCD variants. **a** Percentage (%) of cells showing lamin A aggregation plotted for each CCD 1A variant (mean ± SD, *n* ≥ 3). C2C12 myoblast results are color-coded as follows: skeletal disease-associated variants in red, cardiac in blue, lipodystrophy in yellow, progeria in gray. HEK 293 results are plotted in white or outlined in color if C2C12 myoblast values are zero (e.g., gray and blue for A57P). Inset shows the correlation between HEK 293 cells and C2C12 myoblasts. **b** CCD 1B variants color-coded as above. **c** % aggregation distribution for all CCD variants grouped by phenotype for HEK 293 cells and C2C12 myoblasts with average values outlined in black. Variants with crossover disease are plotted more than once for each phenotype. **d** CCD 2A variants color-coded as above. **e** CCD 2B variants color-coded as above. Asterisks indicate statistically significant increases in aggregation over WT (*p* < 0.05).

Lamin A contains a heptad repeat Coil 1A (aa 28–67) and hendecad repeat patterns Coil 1B (aa 79–222), Coil 2A (240–277), and 2B (aa 278–385) (Supplementary Figures 4 and 5)^8^. Using a GFP-tagged lamin A construct, we overexpressed 24 Coil 1A variants, 47 Coil 1B variants, 12 Coil 2A variants, and 39 Coil 2B variants in HEK 293 cells and C2C12 myoblasts and determined the percentage of GFP-positive cells that exhibit nuclear aggregation (*n* ≥ 3, where *n* equals the number of transfections analyzed) (Supplementary Tables 1–4). Figure 2a, b, d, and e show the percentage of GFP-positive cells containing aggregates for C2C12 myoblasts (color-coded to their disease phenotype(s) compared with HEK 293 results (in white) for each CCD, respectively. Strikingly, 53/70 (76%) of skeletal and 27/60 (45%) of cardiac disease-associated variants formed aggregates in either HEK 293 cells or C2C12 myoblasts. In contrast, 1/7 (14%) of lipodystrophy and 2/13 (15%) of progeria variants aggregated but all also had crossover cardiac or skeletal muscle disease (e.g., R60G, A57P, L59R). Figure 2c highlights this contrast showing the variant aggregation distribution grouped by phenotype (variants with crossover disease are plotted more than once for each phenotype). Overall, there was a good correlation between models for Coil 1A, 1B, and 2B (*R*^2^ = 0.59–0.84) but less so for Coil 2A (*R*^2^ = 0.46), which could be due to the small number of variants compared (Fig. 2a, b, d and e insets). However, several variants that aggregated in HEK 293 cells did not aggregate in C2C12 myoblasts (L52P, A57P, L59R, R60G, I63S, I63N, E203G, R298C, A350P, Q353R, and R377C/H) and vice versa (I46V, R50S, R249W, and R349L), highlighting some variability between overexpression models. We then mapped each variant studied and all variants listed in the UMD and ClinVar databases to locations on the CCD (Supplementary Figures 4 and 5). Although numerous variants are located at ionic interaction sites “e” and “g” important for dimerization, residue “a” important in hydrophobic packing has the most. Site “f”, which has the second most, is located at the surface and these variants may disrupt higher-order assemblies. Together, our CCD results show that most myopathic laminopathies form nuclear aggregates and therefore, this assay might serve as a functional assessment tool towards myopathic variant classification for most variants in the CCDs.

### Aggregation propensity of *LMNA* variants in the IgD

**Fig. 3:**
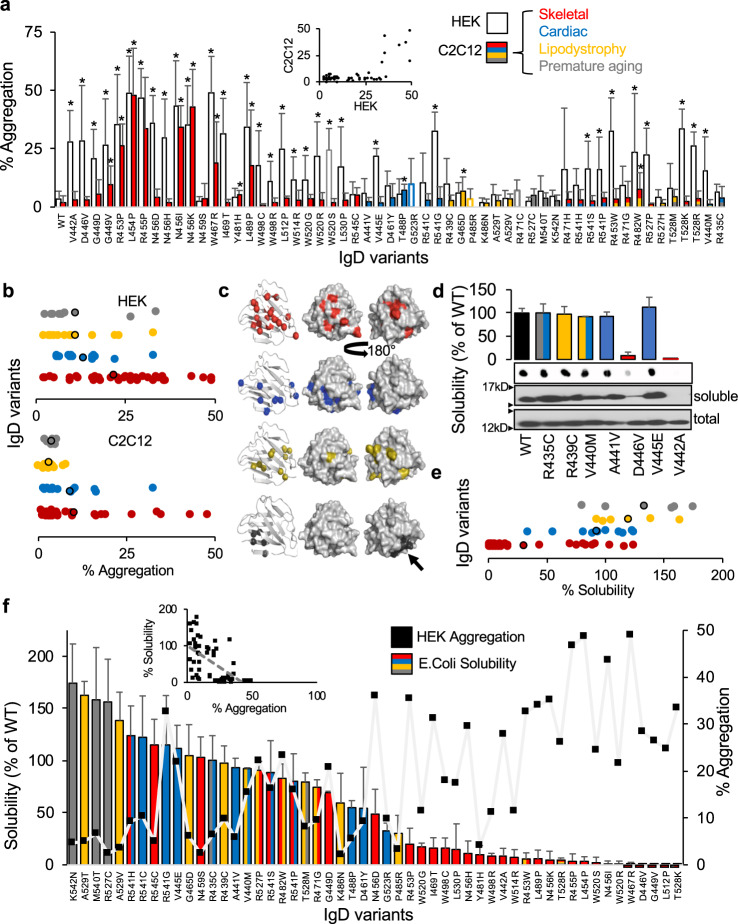
Lamin A aggregation for IgD variants. **a** Percentage (%) of cells showing lamin A aggregation are plotted for each variant (mean ± SD, *n* ≥ 3). C2C12 myoblast variants are color-coded as follows: skeletal disease-associated variants in red, cardiac in blue, lipodystrophy in yellow, progeria in gray. HEK 293 results are plotted in white. Inset shows the correlation between HEK 293 cells and C2C12 myoblasts. **b** % aggregation distribution for all IgD variants grouped by phenotype for HEK 293 cells and C2C12 myoblasts with average values outlined in black. **c** Variants, color-coded as above, are mapped to regions of the IgD structure by disease (red, blue, yellow, and gray balls). The first structure shows the location of each variant followed by surface representations with the last one rotated 180°. Arrow indicates surface patch where progeria-linked variants are located. **d** Representative immunoblots for recombinant IgD variants expressed in *E. coli*. Bars represent relative solubility (% of WT) determined by dot blot for each variant (mean ± SD, *n* ≥ 3). **e** Relative solubility distribution (% of WT determined by dot blot) for all IgD variants grouped by phenotype (*n* ≥ 3) with average values outlined in black. Variants with crossover disease are plotted more than once for each phenotype. **f** Solubility results plotted along with the HEK 293 aggregation results for comparison with inset showing an inverse relationship between aggregation and solubility. Asterisks indicate statistically significant increases in aggregation over WT (*p* < 0.05). *IgD* immunoglobulin-like domain.

The IgD spans amino acids 428–549 and contains 104 reported disease-associated variants (Fig. 1a). We tested aggregation for 56 *LMNA* variants in HEK 293 cells and C2C12 myoblasts and plotted bar graphs (*n* ≥ 3) as described for Coil 1A (Fig. 3a, Supplementary Table 5). Seventeen of 27 (63%) of skeletal and 8/22 (36%) of cardiac variants form aggregates in HEK 293 cells or C2C12 myoblasts. 6/14 (43%) of lipodystrophy and 2/9 (22%) of progeria variants formed aggregates but all had crossover skeletal muscle disease except for G465D. Although there is some overlap between cell models, 23 variants that aggregated in HEK 293 cells did not in C2C12 myoblasts (e.g., V440M) and a few showed small increases in aggregation in C2C12 myoblasts but not in HEK 293 cells (e.g., T488P). Overall and in contrast to the CCDs, IgD variants are more prone to aggregate in HEK 293 cells compared to C2C12 myoblasts, which is underscored in Fig. 3b.

### *LMNA* variants’ effects on IgD domain stability

IgD variants were then mapped to its crystal structure (PDB:1IFR)^9^, a beta-sandwich fold, and color-coded by phenotype (Fig. 3c). Although progeria variants group to one surface suggesting an important protein–protein interaction region (black arrow), lipodystrophy, cardiac, and skeletal variants are scattered throughout. Scharner and co-authors used in silico analyses to show that the cardiac and skeletal myopathy variants are largely buried (Supplementary Table 5); a property commonly associated with loss of stability and also supported by their FoldX computational analysis^25^. Supporting in vitro studies of skeletal disease-associated variants G449V, N456I, and W514R also show IgD destabilization^18,26^. To further validate destabilization as the major cause of aggregation for lamin A IgD variants, we tested 53 variants using an in vitro solubility assay we recently reported where decreased solubility serves as a proxy for protein instability^22^. Figure 3d and Supplementary Figure 1 show representative western and dot blots for seven variants that correlate with our HEK 293 aggregation results; aggregation-prone V442A and D446V showed decreased solubility in contrast to the other five non-aggregating variants that showed no change in solubility compared with WT. Figure 3e and Supplementary Table 5 show the in vitro solubility results for each variant relative to WT (*n* ≥ 3) with direct comparisons with HEK 293 aggregation results plotted in Fig. 3f. Indeed, the inset shows an inverse trend between IgD solubility and lamin A aggregation (*R*^2^ = 0.35) where aggregation-prone IgD variants exhibit less solubility and vice versa. Aggregation results for C2C12 myoblasts also show an inverse trend with IgD solubility (Supplementary Figure 6). Combined, these results help validate misfolding as a major pathogenic mechanism for IgD variants as well as those that do not lead to lamin A aggregation (e.g., P485R, Y481H). Interestingly, several variants have normal solubility but still, aggregate (e.g., V445E, R541G) suggesting more complex misfolding mechanism(s) are involved.

### Aggregation propensity of *LMNA* variants in iPSC-CMs

**Fig. 4:**
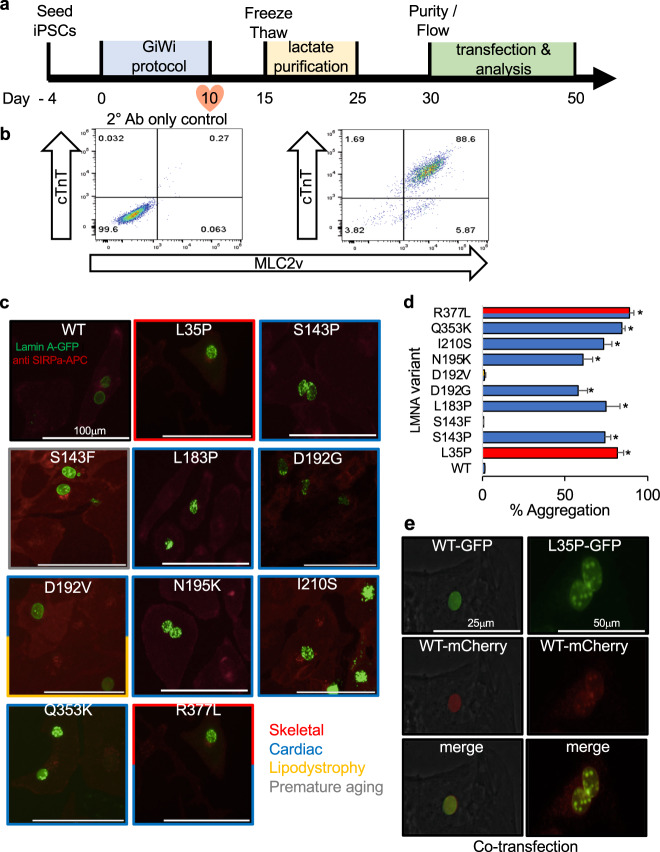
Lamin A nuclear aggregation in iPSC-CMs. **a** A small molecule “GiWi” protocol was used^28^ followed by lactate purification^29^ to generate (**b**) ~90% pure iPSC-CMs (cTnT and MLC2v positive). **c** Representative images of disease-linked lamin A variants (green) overexpressed in SIRPa (cardiomyocyte surface marker) positive cells (red) showing nuclear aggregation^53^. **d** Bars ± SD represents the % of cells showing aggregation for all variants studied color-coded as above (*n* ≥ 3). **e** Representative images of mCherry-WT lamin A (red) co-expressed with GFP-L35P lamin A (green). Asterisks indicate statistically significant increases in aggregation over WT (*p* < 0.05). *GiWi* Gsk3 inhibitor: Wnt inhibitor, *SIRPa* CD47-signal regulatory protein alpha.

Substantiating disease mechanisms in human cardiomyocytes is important for validation and iPSC-based models have been reported for HGPS, FPLD, LGMD1B, and DCM^27^. Although these models recapitulate various nuclear abnormalities and other molecular mechanisms, lamin A aggregation has yet to be shown in human CMs to our knowledge. Further, for a rapid large-scale assay, generating patient iPSCs are not a practical solution and an overexpression system would be advantageous for characterizing a large number of variants. Towards that end, we created iPSC-CMs using a modified “GiWi” protocol^28^ followed by a lactate purification step^29^ to generate ~90% pure cardiomyocytes, based on cardiac troponin T (cTnT) and ventricular myosin light chain-2 (MLC2v) expression as assessed by flow cytometry (Fig. 4a, b). Figure 4c shows representative images for a subset of mostly cardiac disease-linked GFP-tagged lamin A variants overexpressed in SIRPa (CM cell surface marker) positive cells (see Supplementary Figure 3 for examples with a larger field of view). Figure 4d shows the percent aggregation for all variants (*n* ≥ 3) compared with WT (see also Supplementary Table 6). In all cases, variants that aggregate in HEK 293 cell and C2C12 myoblast models also aggregate in human cardiac myocytes. This validates a cell-specific mechanism of protein abnormality associated with cardiomyopathy.

As cardiac and skeletal laminopathies are autosomal dominant diseases, it is important to test aggregation upon co-expression with WT. Figure 4e shows co-assembly and diffuse nuclear expression for both GFP-WT and mCherry-WT lamin A upon co-expression. In contrast, GFP-L35P aggregates and induces mCherry-WT to aggregate upon co-assembly (Fig. 4e). These results show that at least some aggregating disease-linked variants cause dominant-negative effects in human cardiomyocytes further supporting this disease mechanism.

### In silico analysis of Lamin A variants

**Fig. 5:**
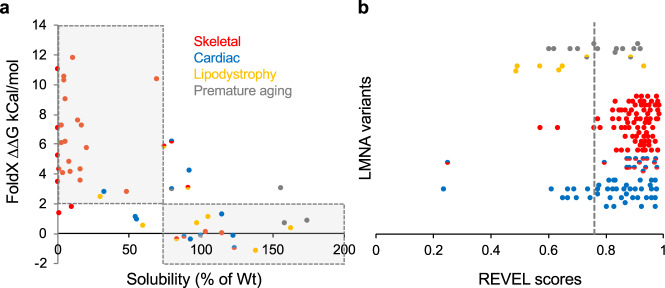
In silico analysis of LMNA variants. **a** Comparison of FoldX and IgD solubility data was reported herein using stability cutoffs of ΔΔG ≤ 2 for FoldX and ≥75% of WT for solubility. The shaded area highlights variants that agree. Color code: cardiac (blue), skeletal (red), lipodystrophy (yellow) and premature aging (gray). **b** Dot plot showing REVEL score distribution of variants with myopathic disease variants (red and/or blue dots) that are not in the gnomAD control population (137 total) compared to all lipodystrophy and/or progeria variants without myopathic crossover disease (yellow and/or gray dots) (20 total). Some myopathic variants also have lipodystrophy/progeria but were not color-coded for simplicity. Variants above the threshold of 0.76 (dashed line) are considered deleterious. *IgD* immunoglobulin-like domain.

Given our relatively large lamin A aggregation and IgD solubility (i.e., deleterious) data sets, we tested the performances of the protein stability prediction program FoldX^23^ and a leading pathogenic prediction program REVEL^24^ for assessing lamin A variants studied herein. Using FoldX ΔΔG values reported by Scharner and co-authors^25^, we found good agreement with our IgD solubility results. Approximately 85% of variants predicted to be destabilizing by FoldX (ΔΔG ≥2) were also less soluble (≤75%) than WT IgD (Fig. 5a).

Next, we analyzed REVEL scores (larger values are more deleterious) for each variant (Supplementary Tables 1–5). To determine an optimal threshold, we searched the large gnomAD reference data sets^30^ to find variant allele frequencies for variants we studied (Supplementary Table 7). Of the myopathic variants that did not aggregate, 15 variants are reported in the gnomAD control population further suggesting they are not causative. Using these as negative controls (Supplementary Tables 1–5) along with 16 pathogenic control variants (P or LP in ClinVar and aggregate in HEK 293 cells and C2C12 myoblasts) we performed a ROC analysis which gave a very good AUC of 93% suggesting that REVEL scores might be predictive of lamin A pathogenicity (Supplementary Figure 7). Using an optimal threshold of 0.76 (where sensitivity equaled specificity) shown in Supplementary Figure 7, we analyzed each variant labeled P/LP by ClinVar or aggregated herein (Supplementary Tables 1–5) and found that 112 variants agreed and 14 did not (~89% accuracy). Interestingly, 7 of the 14 variants that did not agree were lipodystrophy and/or progeria variants labeled P/LP in ClinVar. Further, a dot plot comparing REVEL scores of skeletal/cardiac variants versus lipodystrophy/progeria variants suggests REVEL is a very good classifier of myopathic VUS but not the other types (Fig. 5b).

### Clinical significance of aggregation results

**Fig. 6:**
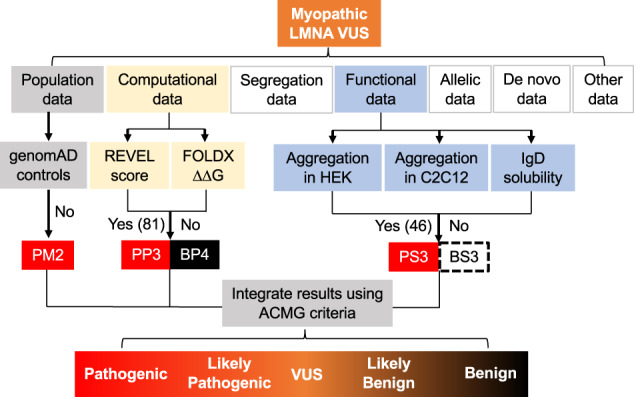
Summary of how our data can support myopathic LMNA variant classification per ACMG guidelines. Flowchart showing the different ACMG classification criteria^21^ and how our data could be incorporated towards classification. PS3 represents functional pathogenic support; the level of which can vary depending on how many of the assays show aggregation. We propose that at least 46 VUS (including those listed as uncertain, not reported, or have conflicting interpretations) meet the criteria for PS3 as they show aggregation in two cell models (Tables S1–S5). The dashed black rectangle for BS3 is to highlight that lack of aggregation is not indicative of benign functional support (particularly for cardiac variants) since there are many other possible pathogenic mechanisms. PP3 and BP4 represent pathogenic and benign computational (REVEL and FoldX) support, respectively with 81 VUS predicted to be pathogenic by REVEL (Tables S1–S5). Lack of variants in the control population from databases like genomAD (PM2) can further strengthen support for the pathogenic classification of LMNA variants in conjunction with the functional and in silico data. *ACMG* American College of Medical Genetics and Genomics, *REVEL* Rare Exome Ensemble Learner, *genomAD* Genome Aggregation Database.

Remarkably, our results reveal that ~80% of the skeletal disease and ~47% of the cardiac disease-linked variants we studied cause significant aggregation, revealing that myopathic laminopathies are predominantly protein misfolding, aggregation-prone diseases (Supplementary Figure 8). Although aggregation for cardiac disease is less, it is a low estimate since most all muscular dystrophies eventually develop cardiomyopathy^31^. In ClinVar, at least 391 *LMNA* missense variants are reported as uncertain with much more undefined or with conflicting interpretations (114 total in our study and listed in Supplementary Tables 1–5). The lack of variant classification represents a significant barrier to providing clinically actionable patient recommendations. Using our nuclear lamin A aggregation results in HEK 293 cells and C2C12 myoblasts as functional evidence of a variant being deleterious, we propose a functional assessment strategy depicted in Fig. 6 adapted from one similarly reported for another protein^32^ to aid in classifying myopathic (and not other types) *LMNA* variants per ACMG guidelines^21^. As shown in Fig. 6, functional assays are just one of several criteria used to help determine pathogenicity and our aggregation assay meets the criteria for functionally supporting pathogenicity (PS3) for myopathic variants (aggregation has been reported by several other groups^10,14,15,19,20^ and a suitable number of controls were used to validate the assay^33^). Further, we performed a ROC-curve analysis using our aggregation data set and HEK 293 cells and C2C12 myoblasts exhibit AUCs of 91% and 77%, respectively, which supports aggregation as being a good predictor for myopathic disease compared to lipodystrophy and progeria laminopathies (Supplementary Figure 9).

Our data supports PS3 assignment per ACMG guidelines for 65 myopathic VUS that significantly increased aggregation in at least one cell type (HEK 293 cells or C2C12 myoblasts). However, 46 increased in both cell types and consequently exhibit stronger functional support for pathogenicity so we propose categorizing these as PS3 (Fig. 6, Supplementary Tables 1–5). We think this more conservative interpretation of the data is preferable until these differences between cell types are understood and more validation studies are done in cardiac and skeletal myocytes. Also of importance in interpreting this assay is that lack of aggregation is not indicative of benign support (BS3) since alternative pathogenic mechanisms may exist and further functional studies are needed, which is highlighted in Fig. 6. We also show that REVEL and FoldX are good predictors of myopathic variants being deleterious and thus can provide computational support for pathogenicity (PP3/BP4) (Fig. 6, Supplementary Tables 1–5). Further, lack of variants in the control population in genomAD also supports pathogenicity (PM2) (Fig. 6). Combined, we show a functional, computational, and population data framework using lamin A aggregation, REVEL/FoldX, and genomAD, respectively that could be applied to nearly all skeletal and most cardiac laminopathic VUS towards pathogenic assessment per ACMG guidelines.

## Discussion

In this study, we use a data-driven approach to establish the formation of misfolded lamin A aggregates as a major consequence of striated muscle laminopathies. This result builds on previous studies showing aggregation of myopathic variants in many different cell types consistent with our results. For example, N195K, E358K, and M371K also aggregate in mouse embryonic fibroblasts^10^ and HeLa cells^19^, R527P in patient fibroblasts^14^, R166P and I210S in COS7 cells^15^ and K97E, L183P, K260N, and Y267C in C2C12 myoblasts^20^. This new insight is important because protein misfolding and aggregation are common disease mechanisms (e.g., neurodegenerative proteinopathies)^17,34,35^ and holds promise for developing therapeutics to restore protein homeostasis and improve clinical outcomes^34,36,37^. Most advances towards the treatment of striated muscle laminopathies involve in vitro strategies targeting signal transduction pathways that are upregulated^38^. Our results show that targeting lamin A misfolding and aggregation either directly or indirectly could also be a promising therapeutic strategy applicable to most striated muscle laminopathies. Further, although *LMNA* variant location (i.e., upstream of the NLS in CCDs but not IgD) is a good predictor of cardiac phenotype severity^39^, it would be intriguing to test, similar to ALS^35^, whether aggregation propensity could be used to predict disease severity for each variant rather than general location alone. This could help in patient management as well as clinical prognostication.

The second key advance from our data-driven approach helps address the gap in variant classification. At present, the number of newly identified variants through genome and exome sequencing^30^ is outpacing their classification, contributing to the excessive number of unclassified rare variants. Understanding the functional impact of variants on disease is essential for classifying variants (i.e., benign, likely benign, uncertain, likely pathogenic, and pathogenic), determining actionability, and advancing personalized medicine^40^. Guidelines have been developed to interpret sequence variation; one being the use of robust in vitro assays as strong evidence towards pathogenic classification^21^ when using an appropriate experimental framework^33^. Several recent studies have taken this approach to systematically analyze a large number of VUS including our previous studies of Long QT Syndrome-associated Kv11.1 variants^22,41^ among several others^32,42,43^. We show lamin A aggregation functionally supports pathogenicity (PS3) for at least 45 myopathic VUS and could be applied to others. Functionally analyzing the ever-growing number of VUS is a big challenge but recent advances in deep mutational scanning combined with workable high-throughput (HTP) functional assays make this goal slightly less daunting^44,45^. Based on our results, *LMNA* may also be amenable to massively parallel functional studies using flow cytometry-based analysis of nuclear protein aggregates^46^.

In silico analyses can be powerful methods of variant classification^21,40^ and gaining rapid structural insight into *LMNA* pathogenicity^25^. We found FoldX (~85% prediction) and REVEL (~89% prediction) to be very good predictors of lamin A pathogenicity. However, 10–15% are still incorrectly classified, and REVEL performed poorly for lipodystrophy and progeria variants. This is likely owing to REVEL being biased towards deleterious changes where many pathogenic variants are not destabilizing (i.e., lipodystrophy and progeria variants) but may disrupt protein-protein interactions for example. As supported by other studies^47^, these computational tools are best used as a complement to functional data analytic tools such as the assays tested here. In addition, we used our results to map each variant to its location within the CCD repeats to reveal any potential phenotype-location or aggregation propensity-location correlations. Overall, variants are over-represented at the ionic interaction residue “e” but largely distributed evenly across all CCD residues making location-based pathogenic predictions unlikely (Supplementary Figure 8). Similarly, myopathic variants mapped to the IgD are scattered but generally buried^25^ (Supplementary Table 5) suggesting IgD instability, which we confirmed here. However, not all destabilized IgD variants caused lamin A aggregation suggesting other mechanisms. For example, IgD is a hotspot for protein-protein interactions and variants could lead to numerous downstream effects without aggregation^48^. Nevertheless, our dataset may be useful for optimizing protein stability and aggregation prediction tools^47^ and gaining further insight into the structural basis of lamin A aggregation.

Mouse models have largely led the way in understanding laminopathies^49^ along with a variety of different overexpression systems showing some mechanistic differences, which are unavoidable limitations in biological research. Our large side-by-side comparison between HEK 293 cells and C2C12 myoblasts shows good agreement for CCD variants but less so for IgD variants, which were more aggregation-prone in HEK 293 cells compared to C2C12 myoblasts. This result shows that aggregation propensity is cell type-dependent. Since, nuclear envelope proteomes differ between tissues^50^, lamin A aggregation may be dependent on cell type-specific partners. Perhaps lamin A aggregation is more common for lipodystrophy and progeria variants when using more appropriate tissue models such as adipocytes and fibroblasts, respectively. Studying aggregation in human myocytes would therefore be particularly useful for characterizing IgD and other variants where there was no agreement between models. Also, aggregation propensity was significant for most variants but less obvious for a few (e.g., I63S, G465D, Y481H) and a more quantitative HTP method would be better to overcome uncertainties due to transfection variability, our relatively small sample size, and user error from manually counting cells^46,51^. Further, the absence of aggregation does not signify a lack of functional support (i.e., BS3) since numerous other mechanisms have been described. This important limitation of this assay is underscored by the skeletal disease-linked variant R545C, which in our study did not aggregate, IgD solubility was similar to WT, and missense variants at that residue are present in the general population. However, R545C was reported as a loss of function using patient myoblasts^52^ highlighting the difficulties of using only heterologous systems or only patient cells, where the loss of function could be attributable to other unknown genomic variables. Cases like these need further functional studies such as iPS cells with isogenic controls. Finally, with the exception of L35P that showed dominant-negative interactions, all of our aggregations results were from variant expression alone and it is possible that aggregation propensity may be dependent on WT co-expression. Moreover, it is possible that aggregation propensity is exaggerated in overexpression models compared to native tissue where expression levels are much lower. With these caveats, it is important to emphasize that aggregation is not a universal functional screen for myopathic laminopathies, but potentially a useful tool to assess uncertain variants. For cardiac variants, in particular, many mechanisms have been described^53^ and while some might just be the downstream effects of lamin A aggregation, ~50% are not and need further study.

Our data-driven approach here demonstrates that lamin A aggregation is a major mechanism underlying striated muscle laminopathies and establishes a functional assay to assist the classification of LMNA rare coding variants.

## Methods

### Variant databases and bioinformatic tools

Laminopathy-associated *LMNA* variants were identified from the Universal Mutational Database (http://www.umd.be/LMNA), ClinVar (https://www.ncbi.nlm.nih.gov/clinvar), and cross-checked for accuracy. Each variant’s disease classification is listed in the Supporting Information (most CMT2B1 designations under “conditions” in ClinVar were not supported and left out of Supplementary Tables). IgD FoldX values were obtained from a study by Scharner and co-authors^25^. ΔΔG values ≥2 were considered destabilizing. REVEL scores were obtained from Ioannidis and co-authors^24^ by entering chromosomal coordinates of variants obtained from ClinVar into the VariED database (varied.cgm.ntu.edu.tw). ROC-curve and AUC analyses were performed using the EPITOOLS web server (epitools.ausvet.com.au). 15 negative controls identified in the control population in genomAD (see Supplementary Tables 1–5) and 16 pathogenic controls (classified P or LP in ClinVar and aggregated in HEK 293 cells and C2C12 myoblasts herein) were used to determine an optimal REVEL score threshold of 0.76 where sensitivity equaled specificity. This threshold was used to decide agreement between REVEL scores and pathogenic predictions.

### DNA plasmid construction

All missense variants were made using the QuikChange II XL kit (Agilent) using primers designed by Integrated DNA Technologies and listed in Supplementary Table 10. The template for mutagenesis was pcDNA3 N195K lamin A (Addgene #32708)^54^, which we mutated back to WT using forward primer CGGGTGGATGCTGAGAACAGGCTGCAGA and reverse primer TCTGCAGCCTGTTCTCAGCATCCACCCG and fully sequenced. Restriction digest analysis was used to test the integrity of all variant constructs, which were then sequenced at the UW-Biotechnology Center. For *E. coli* expression constructs, PCR was used to amplify the IgD (amino acids 435–553) for ligation-independent cloning into a 6X His-tagged pET3 plasmid previously described^22^.

### Cell culture

HEK 293 cells (ATCC) were maintained in Dulbecco’s Modified Eagle Medium (DMEM) containing 1 g/L glucose, 1 mM sodium pyruvate, 4 mM l-glutamine, and 10% FBS. C2C12 myoblasts (MilliporeSigma) were maintained in DMEM containing 4.5 g/L glucose, 1 mM sodium pyruvate, 4 mM l-glutamine, and 10% FBS. iPS cells (DF19-9-11T.H cells from WiCell Stem Bank) were maintained on Matrigel-coated plates using StemFlex media (ThermoFisher) before differentiation. iPSC-CMs older than Day 30 were maintained on Matrigel-coated 6-well plates in RPMI + B27 (ThermoFisher) until transient transfection and imaging experiments. All cells were maintained in a humidified incubator at 37 °C and 5% CO_2_.

### Lamin A immunoblotting

For western blot of full-length GFP-lamin A, HEK 293 cells or C2C12 myoblasts of similar confluence (~90%) were transiently transfected with Lipofectamine 2000 (Invitrogen) using a DNA/lipofectamine ration of 1:3. Cells were grown at 37 °C for 24 h and GFP-lamin A bands were detected by sodium dodecyl sulfate–polyacrylamide gel electrophoresis (SDS-PAGE) analysis. In brief, cells were lysed in HEK 293 lysis buffer (50 mM Tris-HCl pH 7.4, 150 mM NaCl, 1% NP40, protease inhibitor cocktail) and the insoluble material was spun down at 15,000 × *g* for 10 min. Supernatants were mixed with an equal amount of Laemmli sample buffer, separated by SDS/7% PAGE, and detected with 1:1000 anti-GFP-HRP antibody (Santa Cruz Biotechnology #sc-9996-HRP). Blots were derived from the same experiment and processed in parallel.

### IgD solubility assay

Our solubility assay was performed as previously reported^22^. In brief, single colonies of BL21(DE3) cells (New England BioLabs) transformed with each 6X His-tagged variant construct were grown overnight (~18 h) at 37° in 2 ml of auto-induction media. An equal number of cells were harvested, washed (50 mM Tris, 150 mM NaCl, pH 7.5) once, and lysed in *E. coli* lysis buffer (Wash buffer, Cell Lytic B ® (Sigma), and 100 μM phenylmethylsulfonyl fluoride) for 10 min at room temperature. In all, 5 μL of total cell lysate was diluted in 25 μL of wash buffer and added to an equal volume of 2x Laemmli sample buffer before western blot. Soluble protein was collected from the supernatant after a 15,000 × *g* spin for 10 min and diluted in equal amounts of 2× sample buffer for western blot or serially diluted 1:2 for dot blot (1 μL). All samples were boiled for 1–2 mins, run on SDS/12% PAGE, transferred to nitrocellulose paper, and probed with 1:1000 anti-His-HRP antibody (Santa Cruz Biotechnology #sc-8036-HRP). Densitometry was performed using ImageJ (NIH) to quantify immunoblots. For dot blots, one representative row from each serially diluted dot blot was quantified (*n* ≥ 3). Blots were derived from the same experiment and processed in parallel.

### Lamin A aggregation analysis

For HEK 293 cells and C2C12 myoblasts, similarly confluent cells (~80–90%) in 12 or 24 well plates were transfected with 1–2 μg of GFP-lamin A cDNA using a DNA/Lipofectamine 2000 (Qiagen) ratio of 1:3 and grown at 37 °C for 24 h before imaging. For iPSC-CMs, similarly confluent cells (~80–90%) in six-well dishes were transfected with 3 μg of either GFP- WT lamin A or mCherry- WT lamin A (Addgene #55068) using a DNA/ViaFect (Promega) ratio of 1:3. For co-transfections, 1.5 μg of each plasmid was used and grown at 37 °C for 24 h before imaging at ×20 or ×40 magnification using an EVOS FL Imaging System (ThermoFisher). The percentage of cells with lamin A aggregates were obtained by counting ≥100 cells from different fields of view averaged over at least three transfections. All images analyzed were done blinded except for WT to test the quality of the transfected cells (i.e., no cytotoxicity and normal WT-like levels). Counts included cells that had foci of varying sizes and numbers spread throughout the nucleus (the vast majority), some with just one or two large aggregates on one or opposite ends of the nucleus, and some that aggregated into a “honeycomb”-like pattern. For iPSC-CMs, 1:400 APC-anti-CD172a (SIRPa)^55^ (Biolegend #144013) was added directly to cells for 30 min before washing out and imaging. Only cells that were SIRPa positive and/or beating were counted. Examples used to count aggregates are shown in Supplementary Figures 2 and 3. For each set of transfections, WT was also included to check for quality (see large n for WT in Supplementary Tables 1–5), and counting for all variants was done blinded.

### Human iPSC-cardiomyocytes differentiation

19-9-11 hiPSCs were differentiated into hiPSC-CMs using a modified procedure previously described^28^. In brief, hiPSCs maintained on StemFlex/Matrigel were dissociated into single cells and seeded onto Matrigel-coated six-well plates. Cells were cultured for ~5 days in StemFlex medium until they reached 100% confluence. The differentiation (Day 0) started with changing the medium to RPMI + B27 supplement without insulin and 6 µM CHIR99021 (Tocris Bioscience). After 24 h (Day 1), the medium was changed to RPMI + B27 without insulin. After 48 h (Day 3), 5 μM IWP2 (Tocris Bioscience) was added in a combined medium of 1.5 mL of spent medium from the wells and 1.5 ml of fresh RPMI + B27 without insulin. After 48 h (Day 5), the medium was changed to RPMI + B27 without insulin. After 48 h (Day 7), the medium was changed to RPMI + B27 with insulin. The differentiated cells (beating observed ~Day 10) were maintained with medium changes until Day 15 and cryostorage. After the thaw, differentiated hiPSC-CMs were lactate purified in lactate medium (RPMI without glucose, B27 supplement, and 5 mM Sodium DL-lactate) for 10 days^29^. hiPSC-CMs were then maintained in RPMI with B27 supplement. All experiments were done using hiPSC-CMs after Day 30.

### Flow cytometry

Approximately one million iPSC-CMs were dissociated to single cells with 0.25% Trypsin-ethylenediaminetetraacetic acid at 37 °C for 5 min and then pelleted at 1000 rpm for 5 min. The supernatant was removed, and cells were fixed in 1% paraformaldehyde at 37 °C for 10 min in the dark, pelleted, and then resuspended in ice-cold 90% methanol for 30 min. Cells were then pelleted and washed with 3 ml fluorescence-activated cell sorting (FACS) buffer (DPBS without Ca^2+^/Mg^2+^, 0.5% bovine serum albumin, 0.1% Triton X-100, 0.1% NaN_3_) to remove methanol, pelleted again and resuspended in 100 μL FACS buffer. For labeling, 1:200 dilutions of mouse anti-cTnT (ThermoFisher #MA5-12960) and rabbit anti-MLC2v (Proteintech #10906) antibodies were added to the cells in FACS buffer for a final sample volume of 100 μL and incubated at 4 °C overnight (negative control did not receive primary antibody). Cells were then washed in 3 ml of FACS buffer, pelleted, and supernatant was discarded leaving 50 μL. In all, 1:1000 anti-mouse AlexaFluor 568 (Invitrogen #A11031) and 1:1000 anti-rabbit AlexaFluor 488 (Invitrogen #A-11035) secondary antibodies were then added for a final sample volume of 100 μL. Samples were incubated at room temperature in the dark for 30 min, washed in FACS buffer, and resuspended in 300–500 μL FACS buffer for analysis. Data were collected on an Attune Nxt flow cytometer (ThermoFisher) and analyzed with FlowJo. The gating strategy is shown in Supplementary Figure 10.

### Statistical analysis

All aggregation data are presented as mean ± SD. One-way analysis of variance with Dunn’s post hoc test was used to determine differences between variants and WT. *P* < 0.05 was considered statistically significant.

### Ethics statement

This research is in accordance with NIH and the University of Wisconsin-Madison policy for responsible conduction of research. All experiments and procedures have been approved by the University of Wisconsin-Madison policy. The iPSC experiments have been approved by UW Stem Cell Research Oversight (SCRO-1701), and also comply with guidance from NIH Guidelines for Human Stem Cell Research.

### Reporting summary

Further information on research design is available in the Nature Research Reporting Summary linked to this article.

## Supplementary information

Supplementary Information

Reporting Summary

## Data Availability

All variants referenced here can be accessed from ClinVar (www.ncbi.nlm.nih.gov/clinvar) and/or the Universal Mutational Database (www.umd.be/LMNA). ClinVar accession numbers are listed for each variant in the Supplementary Tables. Materials and aggregation data that support the findings of this study are available from the corresponding authors upon reasonable request.

## References

[1] Dittmer TA, Misteli T (2011). The lamin protein family. Genome Biol..

[2] Worman HJ, Bonne G (2007). “Laminopathies”: a wide spectrum of human diseases. Exp. Cell Res..

[3] Bonne G (1999). Mutations in the gene encoding lamin A/C cause autosomal dominant Emery-Dreifuss muscular dystrophy. Nat. Genet..

[4] Fatkin D (1999). Missense mutations in the rod domain of the lamin A/C gene as causes of dilated cardiomyopathy and conduction-system disease. N. Engl. J. Med..

[5] Cao H, Hegele RA (2000). Nuclear lamin A/C R482Q mutation in Canadian kindreds with Dunnigan-type familial partial lipodystrophy. Hum. Mol. Genet..

[6] Eriksson M (2003). Recurrent de novo point mutations in lamin A cause Hutchinson–Gilford progeria syndrome. Nature.

[7] Sandre-Giovannoli AD (2002). Homozygous defects in LMNA, encoding lamin A/C nuclear-envelope proteins, cause autosomal recessive axonal neuropathy in human (charcot-marie-tooth disorder type 2) and mouse. Am. J. Hum. Genet..

[8] Ahn J (2019). Structural basis for lamin assembly at the molecular level. Nat. Commun..

[9] Dhe-Paganon S, Werner ED, Chi Y-I, Shoelson SE (2002). Structure of the globular tail of nuclear lamin. J. Biol. Chem..

[10] Zwerger M (2013). Myopathic lamin mutations impair nuclear stability in cells and tissue and disrupt nucleo-cytoskeletal coupling. Hum. Mol. Genet..

[11] Osmanagic-Myers S, Foisner R (2019). The structural and gene expression hypotheses in laminopathic diseases—not so different after all. Mol. Biol. Cell.

[12] Vigouroux C (2018). Lipodystrophic syndromes due to LMNA mutations: recent developments on biomolecular aspects, pathophysiological hypotheses and therapeutic perspectives. Nucleus.

[13] Broers JLV, Ramaekers FCS, Bonne G, Yaou RB, Hutchison CJ (2006). Nuclear lamins: laminopathies and their role in premature ageing. Physiol. Rev..

[14] Muchir A (2004). Nuclear envelope alterations in fibroblasts from patients with muscular dystrophy, cardiomyopathy, and partial lipodystrophy carrying lamin A/C gene mutations. Muscle Nerve.

[15] Cowan J, Li D, Gonzalez-Quintana J, Morales A, Hershberger RE (2010). Morphological analysis of 13 LMNA variants identified in a cohort of 324 unrelated patients with idiopathic or familial dilated cardiomyopathy. Circ. Cardiovasc. Genet..

[16] Tienen FHJvan (2019). Assessment of fibroblast nuclear morphology aids interpretation of LMNA variants. Eur. J. Hum. Genet..

[17] Baets GD, Doorn LV, Rousseau F, Schymkowitz J (2015). Increased aggregation is more frequently associated to human disease-associated mutations than to neutral polymorphisms. Plos Comput. Biol..

[18] Dutta S (2018). Skeletal muscle dystrophy mutant of lamin A alters the structure and dynamics of the Ig fold domain. Sci. Rep. UK.

[19] Hübner S, Eam JE, Wagstaff KM, Jans DA (2006). Quantitative analysis of localization and nuclear aggregate formation induced by GFP‐lamin A mutant proteins in living HeLa cells. J. Cell Biochem..

[20] Bhattacharjee P, Dasgupta D, Sengupta K (2017). DCM associated LMNA mutations cause distortions in lamina structure and assembly. Biochim. Biophys. Acta Gen. Subj..

[21] Richards S (2015). Standards and guidelines for the interpretation of sequence variants: a joint consensus recommendation of the American College of Medical Genetics and Genomics and the Association for Molecular Pathology. Genet. Med..

[22] Anderson CL (2020). A rapid solubility assay of protein domain misfolding for pathogenicity assessment of rare DNA sequence variants. Genet. Med..

[23] Schymkowitz J (2005). The FoldX web server: an online force field. Nucleic Acids Res..

[24] Ioannidis NM (2016). REVEL. An ensemble method for predicting the pathogenicity of rare missense variants. Am. J. Hum. Genet..

[25] Scharner J, Lu H, Fraternali F, Ellis JA, Zammit PS (2014). Mapping disease‐related missense mutations in the immunoglobulin‐like fold domain of lamin A/C reveals novel genotype–phenotype associations for laminopathies. Proteins Struct. Funct. Bioinform..

[26] Dialynas G (2015). Myopathic lamin mutations cause reductive stress and activate the Nrf2/Keap-1 pathway. Plos Genet..

[27] Crasto S, Di Pasquale E (2018). Induced pluripotent stem cells to study mechanisms of laminopathies: focus on epigenetics. Front. Cell Dev. Biol..

[28] Lian X (2013). Directed cardiomyocyte differentiation from human pluripotent stem cells by modulating Wnt/β-catenin signaling under fully defined conditions. Nat. Protoc..

[29] Tohyama S (2013). Distinct metabolic flow enables large-scale purification of mouse and human pluripotent stem cell-derived cardiomyocytes. Cell Stem Cell.

[30] Karczewski KJ (2020). The mutational constraint spectrum quantified from variation in 141,456 humans. Nature.

[31] Peretto, G. et al. Cardiac and neuromuscular features of patients with LMNA-related cardiomyopathy. *Ann. Intern. Med*. **171**, 458–463. 10.7326/M18-2768 31476771

[32] Hong JH (2020). Functional characterization guides classification of novel BAP1 germline variants. NPJ Genom. Med..

[33] Brnich SE (2019). Recommendations for application of the functional evidence PS3/BS3 criterion using the ACMG/AMP sequence variant interpretation framework. Genome Med..

[34] Sweeney P (2017). Protein misfolding in neurodegenerative diseases: implications and strategies. Transl. Neurodegener..

[35] Wang Q, Johnson JL, Agar NYR, Agar JN (2008). Protein aggregation and protein instability govern familial amyotrophic lateral sclerosis patient survival. Plos Biol..

[36] Hipp MS, Park S-H, Hartl FU (2014). Proteostasis impairment in protein-misfolding and -aggregation diseases. Trends Cell Biol..

[37] Liguori L (2020). Pharmacological chaperones: a therapeutic approach for diseases caused by destabilizing missense mutations. Int. J. Mol. Sci..

[38] Schreiber KH, Kennedy BK (2013). When lamins go bad: nuclear structure and disease. Cell.

[39] Captur G (2018). Lamin mutation location predicts cardiac phenotype severity: combined analysis of the published literature. Open Heart.

[40] Peterson TA, Doughty E, Kann MG (2013). Towards precision medicine: advances in computational approaches for the analysis of human variants. J. Mol. Biol..

[41] Anderson CL (2014). Large-scale mutational analysis of Kv11.1 reveals molecular insights into type 2 long QT syndrome. Nat. Commun..

[42] Woods NT (2016). Functional assays provide a robust tool for the clinical annotation of genetic variants of uncertain significance. Npj Genom. Med..

[43] Wiltshire T (2019). Functional characterization of 84 PALB2 variants of uncertain significance. Genet. Med..

[44] Starita LM (2017). Variant interpretation: functional assays to the rescue. Am. J. Hum. Genet..

[45] Weile J (2017). A framework for exhaustively mapping functional missense variants. Mol. Syst. Biol..

[46] Whiten DR (2016). Rapid flow cytometric measurement of protein inclusions and nuclear trafficking. Sci. Rep..

[47] Khan S, Vihinen M (2010). Performance of protein stability predictors. Hum. Mut..

[48] Dittmer TA (2014). Systematic identification of pathological lamin A interactors. Mol. Biol. Cell.

[49] Stewart CL, Kozlov S, Fong LG, Young SG (2007). Mouse models of the laminopathies. Exp. Cell Res..

[50] Korfali N (2012). The nuclear envelope proteome differs notably between tissues. Nucleus.

[51] Scotter EL, Narayan P, Glass M, Dragunow M (2008). High throughput quantification of mutant huntingtin aggregates. J. Neurosci. Methods.

[52] Kandert S, Wehnert M, Müller CR, Buendia B, Dabauvalle M-C (2009). Impaired nuclear functions lead to increased senescence and inefficient differentiation in human myoblasts with a dominant p.R545C mutation in the LMNA gene. Eur. J. Cell Biol..

[53] Crasto S, My I, Di Pasquale E (2020). The broad spectrum of LMNA cardiac diseases: from molecular mechanisms to clinical phenotype. Front. Physiol..

[54] Gilchrist S (2004). Altered protein dynamics of disease-associated lamin A mutants. BMC Cell Biol..

[55] Dubois NC (2011). SIRPA is a specific cell-surface marker for isolating cardiomyocytes derived from human pluripotent stem cells. Nat. Biotechnol..

