# Supplementary material for: Most myopathic lamin variants aggregate: a functional genomics approach for assessing variants of uncertain significance
Source: NPJ Genom Med. 2021 Dec 3;6:103. doi: 10.1038/s41525-021-00265-x (PMC8642518; doi:10.1038/s41525-021-00265-x)
Supplement: Supplementary file 1 — Supplementary Information [file 41525_2021_265_MOESM1_ESM.pdf]

a

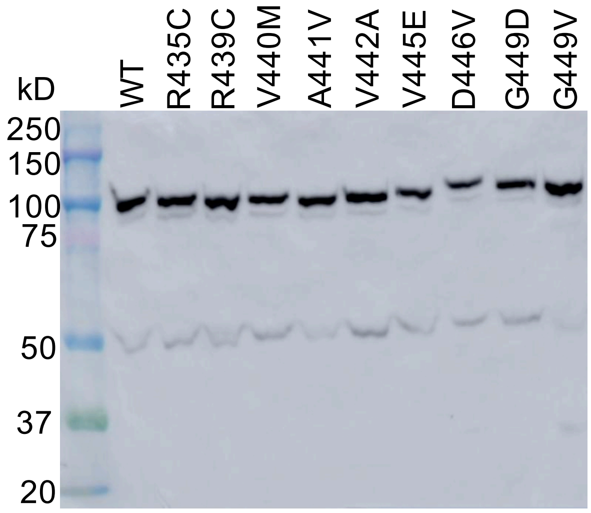

b

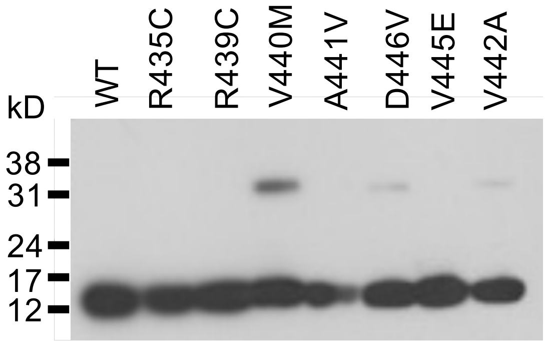

c

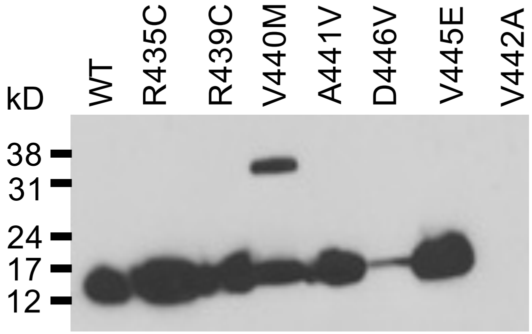

Supplementary Figure 1. Representative immunoblots. (**a**) un-cropped immunoblot of GFP-lamin A variants expressed in HEK 293 cells from Fig. 1D. (**b,c**) un-cropped immunoblots of His-tagged IgD variants expressed in *E.coli* from Fig. 3D for the total and soluble fractions, respectively. All blots derive from the same experiment and were processed in parallel.

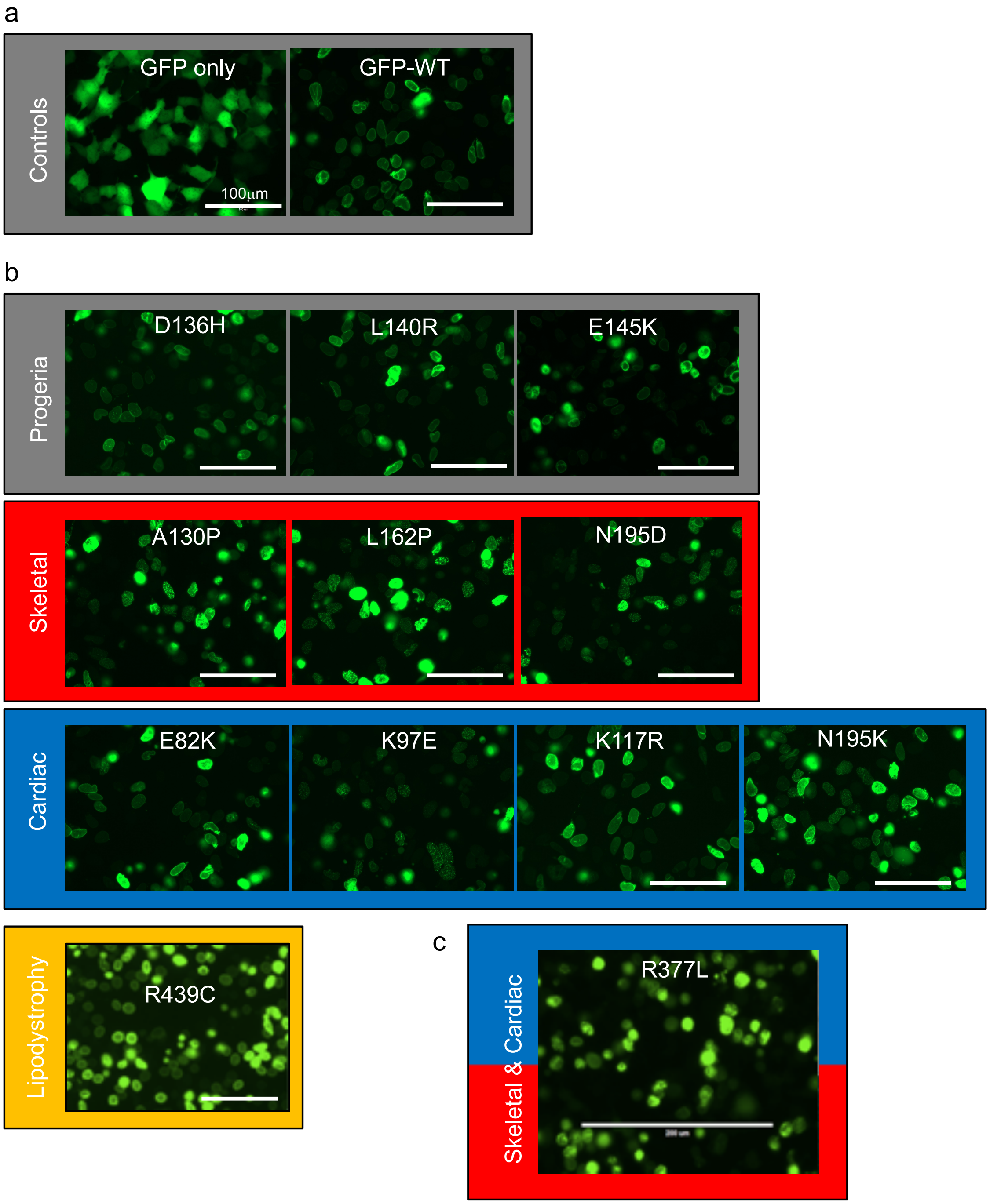

Supplementary Figure 2. Representative HEK 293 images used for determining % of cells with nuclear lamin aggregates. (**a**) GFP alone showing uniformly diffuse expression throughout the cell compared to GFP-WT-lamin A showing diffuse nuclear expression. (**b**) Examples of each class of variant with some showing lamin aggregation (K97E, A130P, L162P, N195D, and N195K). (**c**) For some variants (e.g. R377L), "honeycomb"-like mislocalization was also observed, which were also counted as aggregation for simplification.

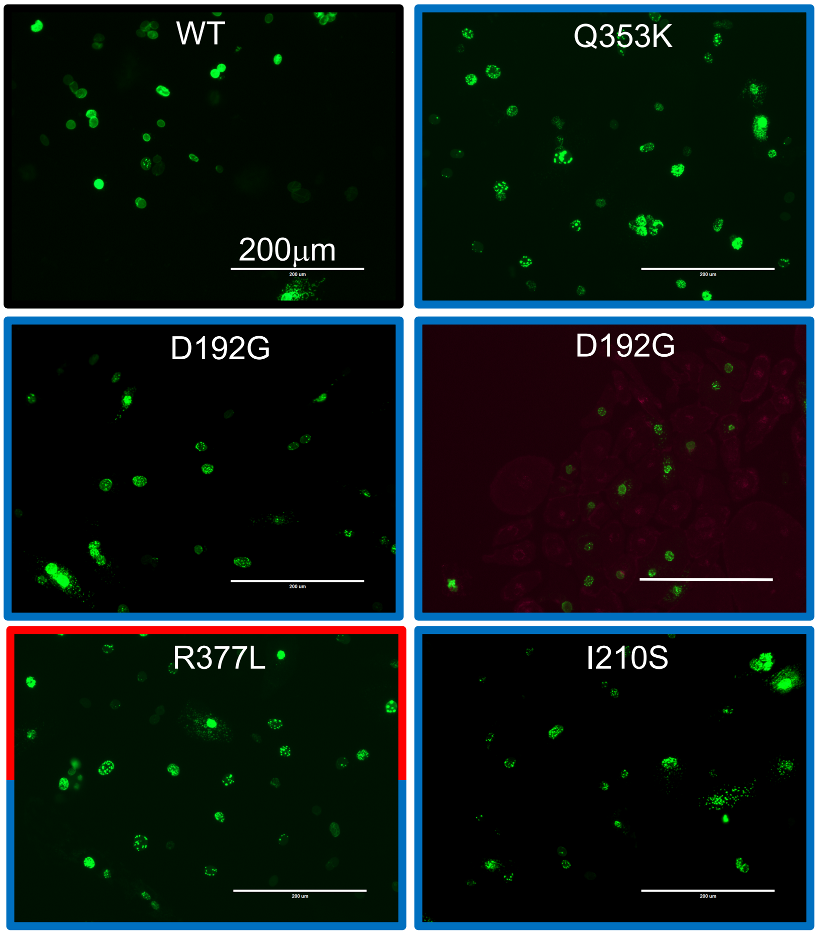

Supplementary Figure 3. Representative iPSC-CM images used for determining % of cells with nuclear lamin A aggregates. D192G with and without SIRPa (cardiomyocyte cell surface marker) staining.

a

a	b	c	d	e	f	g
R28G	Q36L	E37K	E31K	K32E	E33D	D34N
R28Q	A43E	V44F	L38H	N39D	E33G	R41C
R28W	A43T	E65G	Y45C	N39H	D47H	R41P
L35P	R50C		L52P	N39I	D47N	R41L
L35V	R50H		L52V	N39S	D47Y	R41H
V49L	R50L		L59R	N39Y	T54M	R41S
I63N	R50P		L59V	I46L	L61P	R48L
I63S	R50S			I46N		R48P
I63T	A57P			I46V		E55G
	T64N			E53G		E55K
				E53V		R62C
				R60C		R62G
				R60G		
				R60P		
				E67C		

b

a	b	c	d	e	f	g	h	i	j	k
L85H	A100P	R101H	Y81C	E82K	A79D	E84D	Y211C			
L85P	N142D	R101P	Y81H	R89C	K90E	E84K	H222P			
L85R	H163Q	K108E	Y81F	R89H	K97E	T91I	H222Y			
L92F	V191M	E115V	A88G	R89L	K97T	E112G				
L92V	R216C	A129D	A88V	A96S	L104R	E112K				
R99P	R216H	D136E	L102P	Q103R	E111K	E112V				
R99S		D136G	L102Q	R110H	E111Q	R119P				
L148P		D136H	V109E	R110P	A118S	D126E				
L162P		S143F	K123R	R110S	G125A	R133L				
L183P		S143P	A130D	K117R	G125S	R133P				
R190P		T150A	A130P	E124G	A132P	R133Q				
R190Q		T150I	L137P	E138K	A146T	L140P				
R190W		T150P	L137V	E145K	L188R	L140Q				
L204Q		T157R	K144T	E159K	N195D	L140R				
L204R		K171N	A151P	R166P	N195K	A147P				
L204V		D192G	L165V	R166Q	E202K	E161K				
L215V		D192V	E186K	R166W	R220C	Q168H				
L215P		F206L	M200V	L215P	R220H	R189P				
			T218P	K219N		R189Q				
				K219T		R189W				
						E203G				
						E203K				
						E203V				
						I210S				
						R221C				
						R221P				

Supplementary Figure 4. CCD 1 variant location. (**a**,**b**) All laminopathic missense variants listed in Universal Mutational Database and ClinVar for CCD 1A and 1B, respectively. Color code: cardiac (blue), skeletal (red), lipodystrophy (yellow) and premature aging (gray), condition not provide in ClinVar (clear). ClinVar lists R99S, K117R, and R133Q as having all possible conditions, which is likely incorrect. Those not categorized in the UMD were left clear.

a

a	b	c	d	e	f	g	h	i	j	k
L245P	Q246K	E247K	L248P	R249G	A250V	E262K	L263M	R275G	D243Y	A244T
Y267C	Q246R	A269P	Y259D	R249Q	A250T		L263P		D254E	A244V
Y267H	E257A		Y259H	R249W			H252N		D254N	T266A
	S268P		K270N	K260E			H252P		Q276P	S277P
				K260N						
				L271P						

b

a	b	c	d	e	f	g	h	i	j	k
A278P	E279K	A287V	A288G	S282N	N283S	L284P	L327V		R329H	
A278T	D300G	Q294P	S295P	H289P	E290K	E291K			R329C	
V285L	D300H	T333S	S295L	R296C	I297S	R298C			R329G	
L292P	D300N	E347K	L302P	S303P	I297V	R298L			R329P	
L929R	A339E	Q354K	S334N	E317K	K311R	R298P			R329S	
I299M	A339V	Q354R	M348I	R335Q	A318T	Q312H				
I299T	Q353K	E361K	L369P	R335W	R336Q	S326T				
I299V	Q353R	E361Q	Y376H	E342K	R343Q	E358G				
L306P	E381A	A375D	E383K	R349L	R343W	E358K				
L306R		A375G		R349W	A350P	E372D				
L306V		A375T		L356R	D357A	E372K				
R331Q		G382R		D370E	D357H	L379F				
R331P		G382V		R377C	D357Y	L379V				
R331W				R377H	D357V					
I373D				R377L	D364N					
I373F				E384D	M371K					
I373M					M371V					
L380S										

Supplementary Figure 5. CCD 2 variant location. (**a,b**) All laminopathic missense variants listed in Universal Mutational Database and ClinVar for CCD 2A and 2B, respectively. Color code: cardiac (blue), skeletal (red), lipodystrophy (yellow) and premature aging (gray), condition not provide in ClinVar (clear). ClinVar lists A250V, T154A, A318V, R329G, R329S, R335W, R343W and R349W as having all possible conditions, which is likely incorrect. Those not categorized in the UMD were left clear.

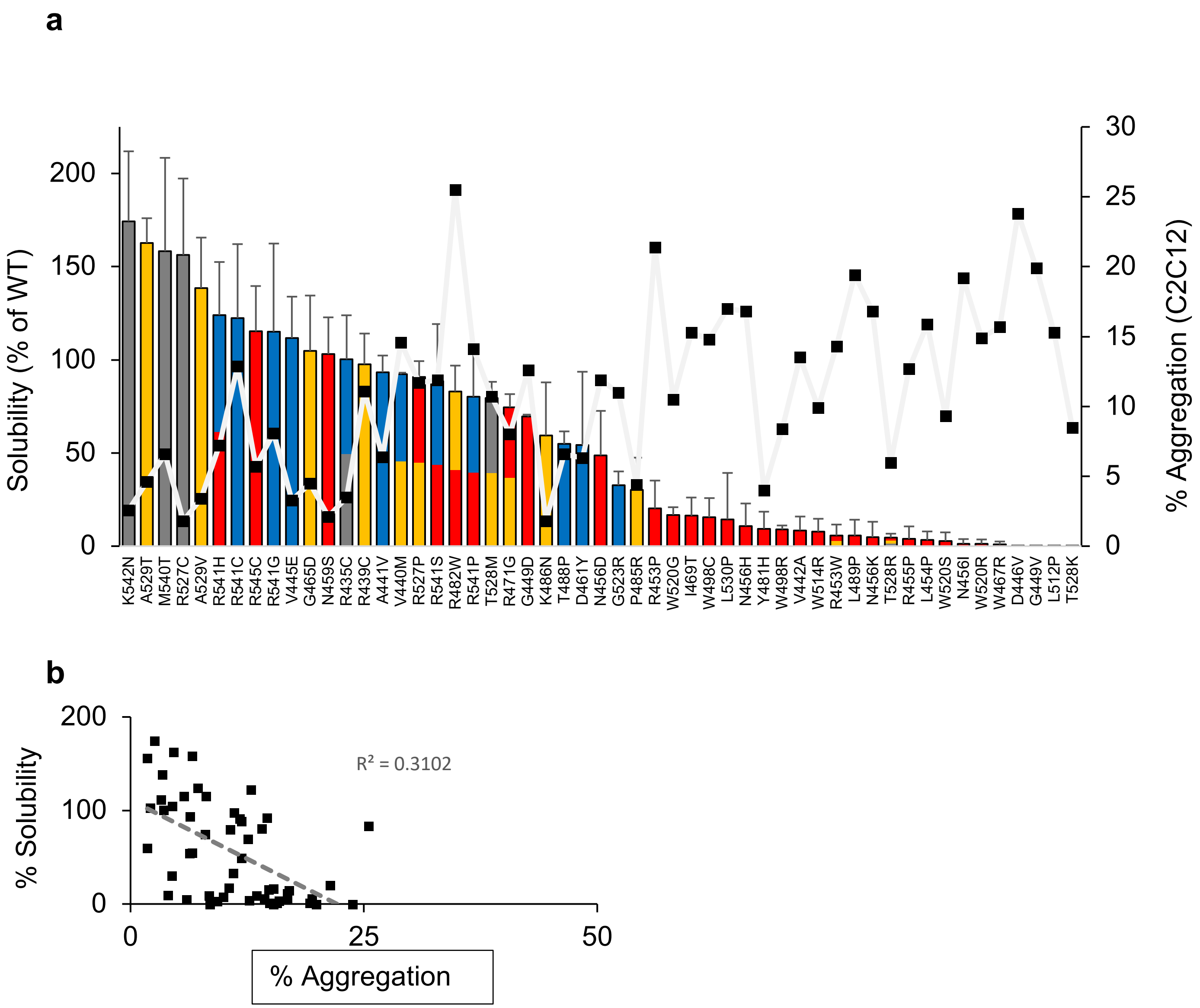

Supplementary Figure 6. Lamin IgD variant aggregation and solubility correlation. (**a**) Relative solubility (% of WT determined by dot blot) for all variants (n≥3) plotted along with the C2C12 myoblast aggregation results. Color code: cardiac (blue), skeletal (red), lipodystrophy (yellow) and premature aging (gray). (**b**) Inverse relationship between aggregation and solubility. Abbreviations: Immunoglobulin-like domain (IgD).

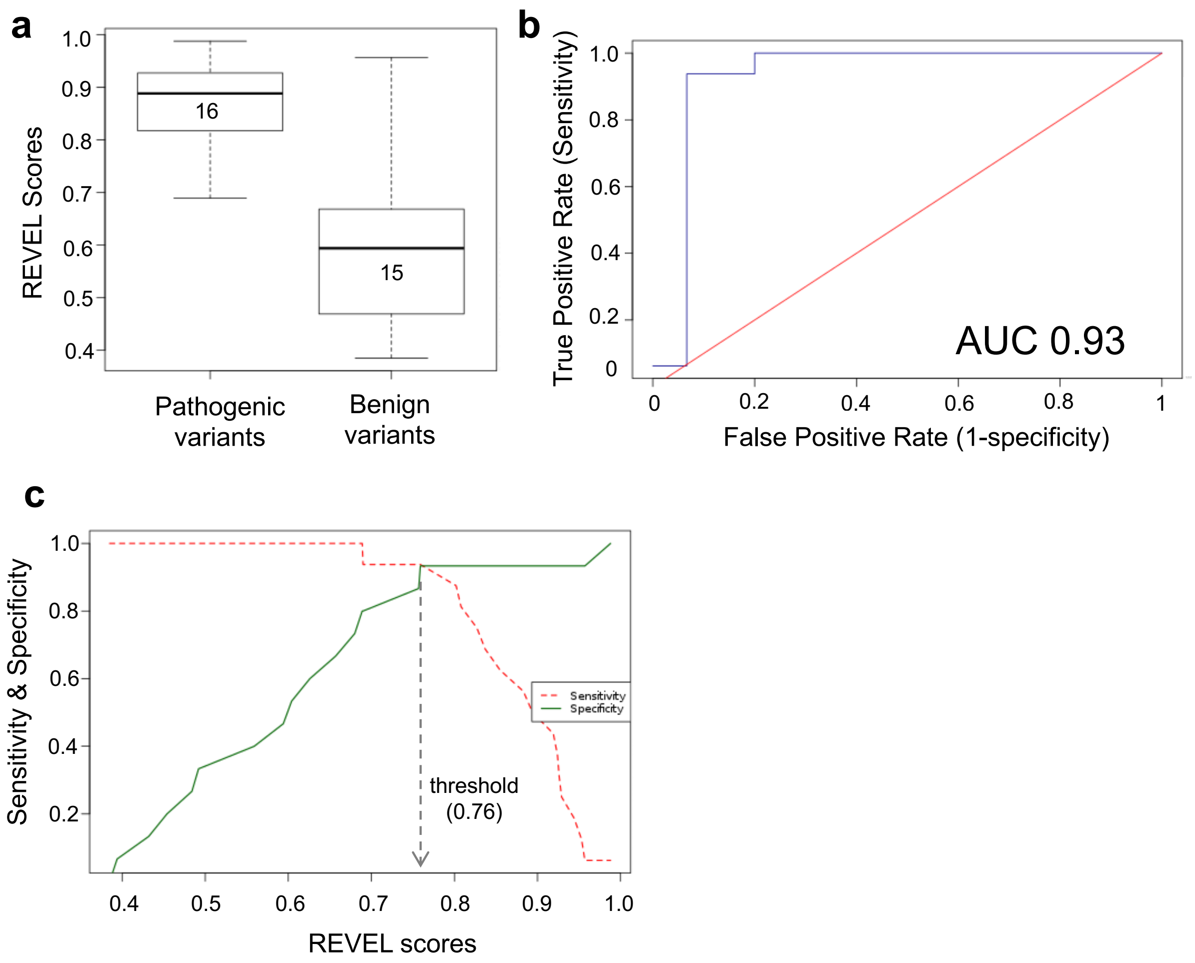

Supplementary Figure 7. REVEL score analysis of lamin A variants studied. (**a**) REVEL scores for 16 pathogenic variants were compared to 15 benign variants to generate (**b**) ROC-curve (AUC = 93%, 95% CI 0.806-1) with optimal threshold for pathogenicity shown in the two graph ROC curve (**c**). Red line in (**b**) indicates no discrimination between groups. Abbreviations: receiver operating characteristic (ROC), area under the curve (AUC), confidence interval (CI).

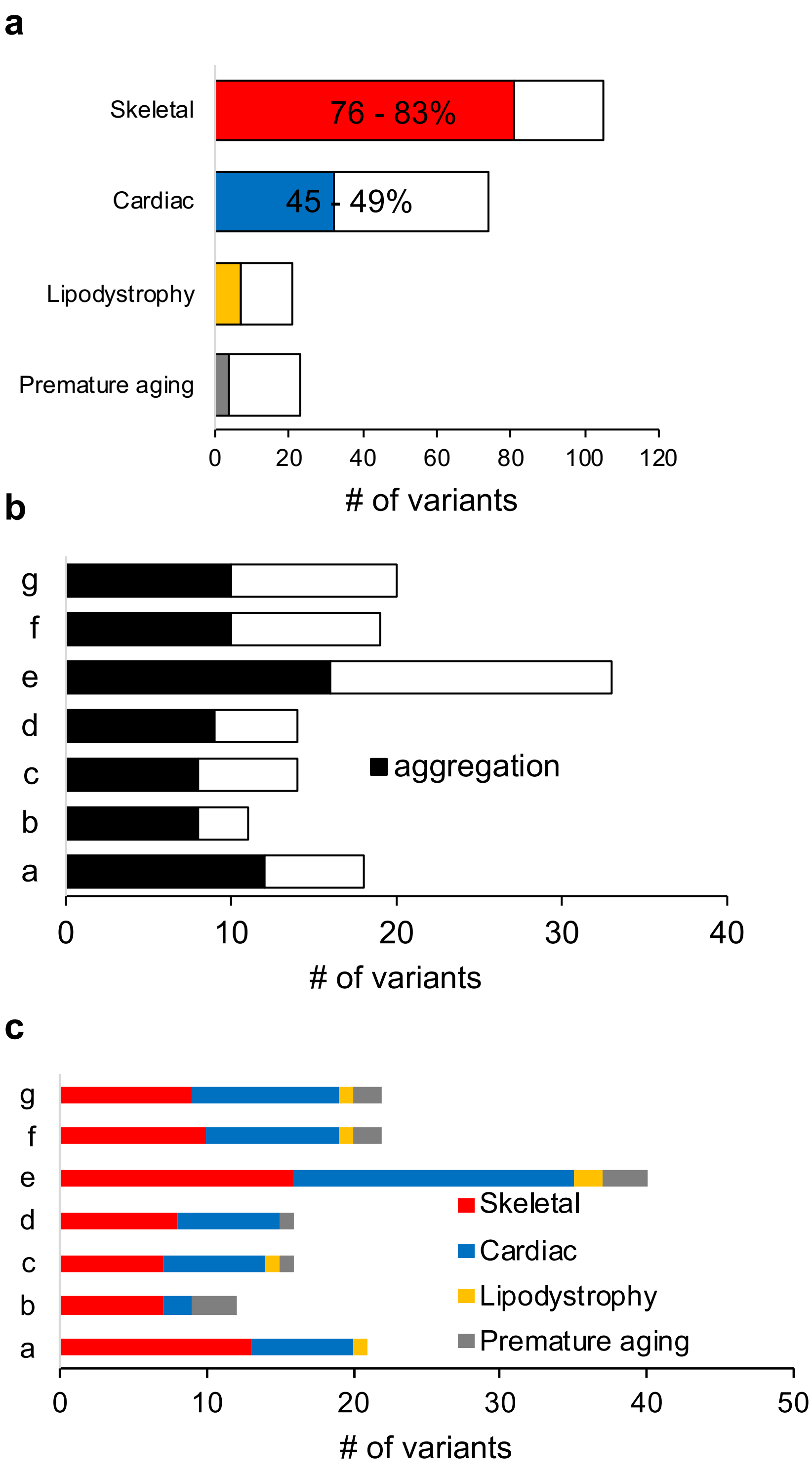

Supplementary Figure 8. Variant and aggregation distribution. (a) Total number of variants studied with the number that aggregate shown with filled bars. Range is before and after subtracting proposed benign or likely benign variants from allele frequency analysis. (**b**) Variant aggregation distribution by CCD location with the number that aggregate shown with filled bars. (**c**) CCD location for all assayed variants by disease.

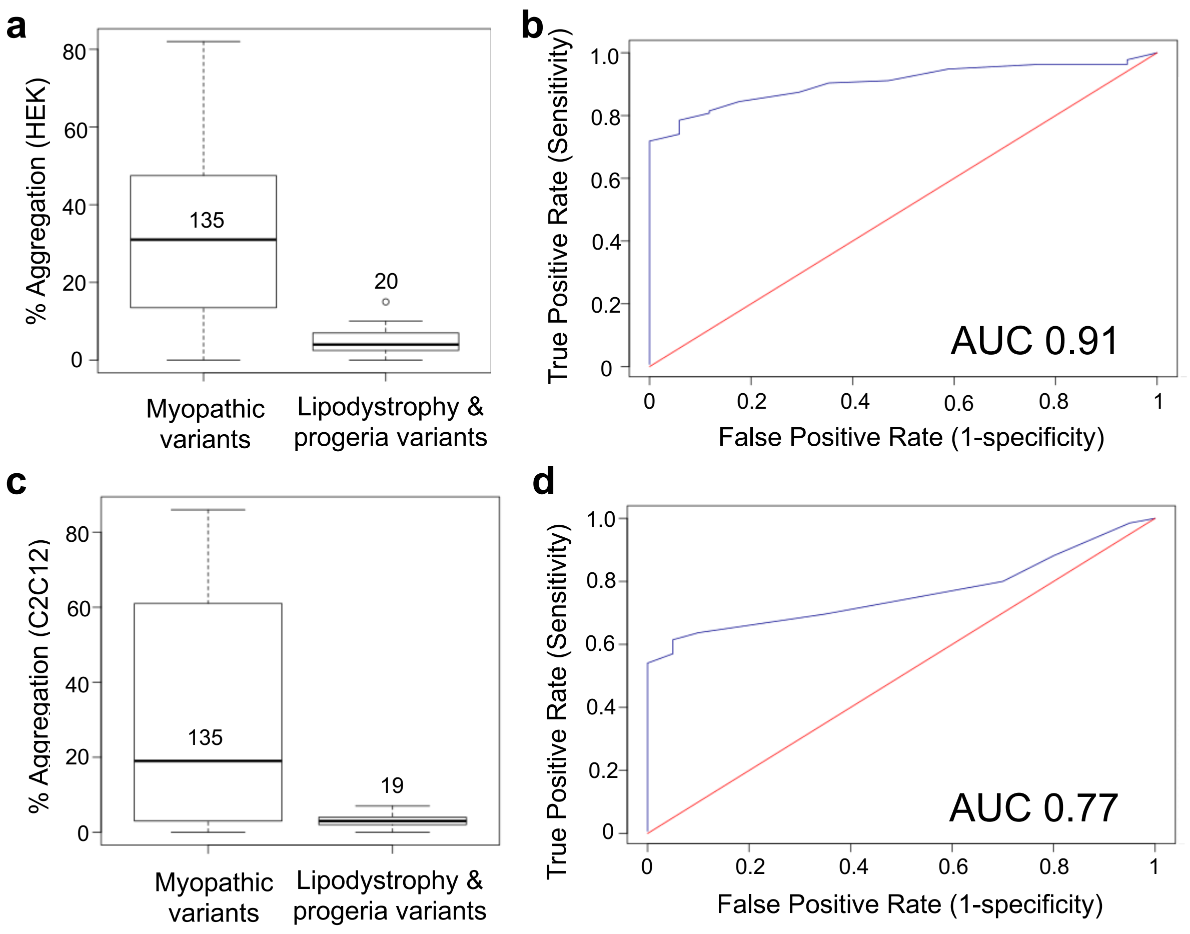

Supplementary Figure 9. ROC-AUC analysis to test aggregation as a classifier of myopathic versus lipodystrophy/progeria laminopathies. Variants with skeletal and/or cardiac disease which were not present in the gnomAD control population (135 total for HEK 293 cells and C2C12 myoblasts) were compared to all variants with lipodystrophy and/or progeria without myopathic crossover disease (20 total for HEK 293, 19 total for C2C12 myoblasts). (**a**) Box plot of all variants used to generate the ROC-curve shown in (**b**) with an AUC of 91% (CI 0.86-0.95) for HEK 293 cells. (**c**) Box plot of all variants used to generate the ROC-curve shown in (**d**) with and AUC of 77% (CI 0.69-0.85) for C2C12 myoblasts. Red lines indicate no discrimination between groups. Abbreviations: receiver operating characteristic (ROC), area under the curve (AUC), confidence Interval (CI).

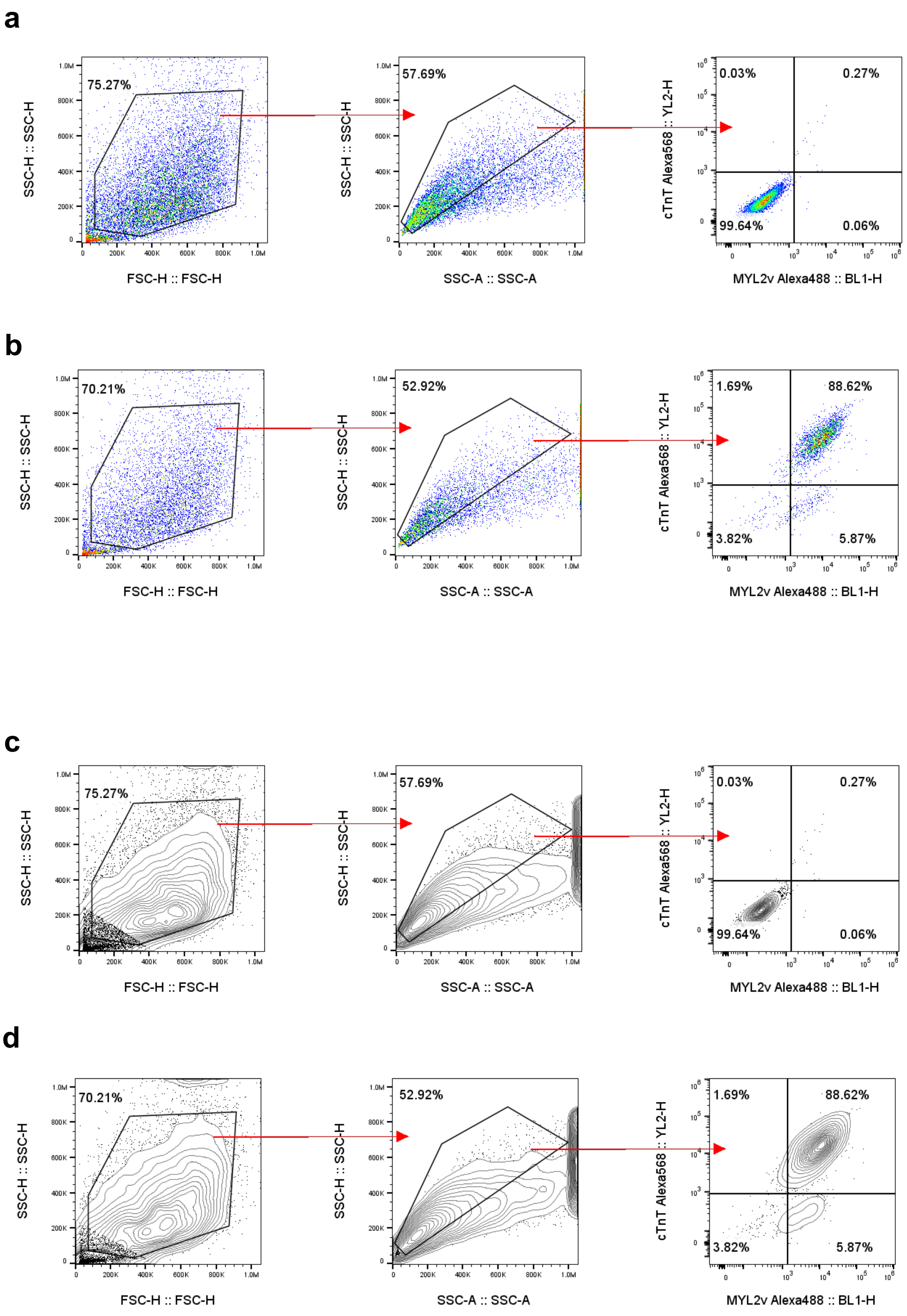

Supplementary Figure 10. Gating strategy for flow cytometry analysis. A cell population was selected (SSC-H vs FSC-H), then gated for singlets (SSC-H vs SSC-A) and then gated for cTnT and MYL2v positive cells to determine the percentage of cardiomyocytes. Dot plots show (**a**) secondary antibody only control and (**b**) primary and secondary antibody. (**c**) and (**d**) is the same data shown as gradient plots, respectively.

Coil 1A	Accession	CM	SM	Lipo	PA	Location	ClinVar	HEK ± SD	N	C2C12 ± SD	N	REVEL	Function / Prediction
WT		-	-	-	-	-	-	3 ± 3	57	2 ± 3	31	-	
E33D*	VCV000066965		+			f	U	39 ± 38	5	41 ± 8	3	0.573	PS3/BP4
E33G	VCV000066962		+			f	UD	42 ± 38	5	40 ± 8	3	0.909	PS3/PP3
L35V	VCV000066761		+			a	UD	58 ± 21	3	61 ± 28	3	0.849	PS3/PP3
L35P	VCV000066765		+			a	UD	82 ± 10	3	65 ± 26	3	0.931	PS3/PP3
N39D	not listed		+			e	UD	24 ± 11	3	51 ± 28	4	0.938	PS3/PP3
N39S	VCV000066791		+			e	P	40 ± 32	5	59 ± 27	3	0.883	
N39Y	VCV000066788		+			e	UD	46 ± 45	5	58 ± 28	3	0.985	PS3/PP3
R41C	not listed		+			g	UD	46 ± 43	5	58 ± 29	3	0.945	PS3/PP3
R41S	VCV000648598		+			g	P	44 ± 46	5	55 ± 25	3	0.924	
A43T	VCV000066799		+			b	UD	32 ± 31	5	22 ± 12	3	0.966	PS3/PP3
Y45C	VCV000066807	+	+			d	UD	43 ± 42	5	42 ± 17	3	0.876	PS3/PP3
I46V	VCV000066815		+			e	LP	6 ± 2	3	37 ± 27	3	0.869	
D47H	VCV000066822		+			f	U	0 ± 0	3	1 ± 1	3	0.911	PP3
R50H	VCV000519299		+			b	U	4 ± 4	3	0 ± 0	3	0.88	PP3
R50P	VCV000066840		+			b	UD	38 ± 31	4	68 ± 8	3	0.87	PS3/PP3
R50S	VCV000066835		+			b	UD	6 ± 2	3	38 ± 15	3	0.95	PP3
L52P	VCV000066847	+				d	UD	23 ± 24	6	6 ± 4	3	0.975	PP3
E53V	VCV000066852	+				e	UD	7 ± 4	6	1 ± 1	3	0.979	PP3
A57P	VCV000014506	+			+	b	P	12 ± 9	7	0 ± 0	3	0.829	
L59R	VCV000018457	+			+	d	P	20 ± 14	5	1 ± 1	3	0.958	
R60G	VCV000014479	+		+		e	P	15 ± 9	5	1 ± 2	3	0.699	
I63S	VCV000066872		+			a	UD	8 ± 5	7	1 ± 1	3	0.847	PP3
I63N	VCV000066871		+			a	UD	11 ± 6	6	2 ± 2	3	0.935	PP3
E65G	VCV001074127		+			c	P	8 ± 7	6	1 ± 1	3	0.907	

Supplementary Table 1. Properties of Coil 1A variants. Variants studied are color-coded by disease-association with cardiac muscle disease (blue), skeletal muscle disease (red), lipodystrophy (yellow), premature aging (gray), or more than one (e.g. Y45C) as listed in the UMD-LMNA database. Accession numbers from ClinVar. Asterisk indicates linkage to Charcot-Marie Tooth Disease (Goizet et al. J Med Genet 2004). Location indicates residue position on CCD (see helical wheel diagram in Fig. 1). ClinVar abbreviations designate classifications as of July 2021: pathogenic (P), likely pathogenic (LP), uncertain (U), undetermined (not provided or **conflicting interpretation**) (UD). HEK 293 and C2C12 myoblast columns lists % of cells that aggregate (mean ± SD) by ANOVA-Dunn's post hoc test ($P < 0.05$). REVEL column shows scores for each variant filled blue if above the 0.76 threshold (see Supplementary Figure 7) or green if below. Function/Prediction column lists ACMG supporting classifiers for myopathic variants based on our results that are listed as uncertain or undetermined in ClinVar (shaded gray). PS3 represents pathogenic supporting (functional data) if both HEK 293 cells and C2C12 myoblasts show increased aggregation. PP3 represents pathogenic supporting (computational data) if REVEL score is above the threshold.

Coil 1B	Accession	CM	SM	Lipo	PA	Location	ClinVar	HEK ± SD	N	C2C12 ± SD	N	REVEL	Function / Prediction
WT		-	-	-	-	-	-	3 ± 3	57	1.6 ± 3	31	-	
E82K	VCV000066882	+				i	P	4 ± 5	4	4 ± 4	3	0.894	
L85P	not listed		+			a	UD	78 ± 6	3	33 ± 28	3	0.974	PS3/PP3
L85R	VCV000014480	+				a	P	8 ± 9	7	3 ± 3	3	0.975	
R89C	VCV000066883		+			e	UD	78 ± 30	4	79 ± 30	4	0.907	PS3/PP3
R89L	VCV000066884	+	+			e	P	61 ± 41	4	74 ± 26	4	0.944	
L92F	VCV000066885	+		+		a	LP	23 ± 22	5	5 ± 6	4	0.884	
K97E	VCV000066886	+				f	UD	51 ± 38	5	60 ± 22	4	0.84	PS3/PP3
R101P	VCV000066889	+	+			c	U	70 ± 7	3	78 ± 22	4	0.917	PS3/PP3
L102Q	not listed		+			d	UD	3 ± 3	4	6 ± 6	3	0.905	PP3
R110H	VCV000522979	+				e	UD	3 ± 2	3	1 ± 1	3	0.604	**neg control** / BP4
R110S	VCV000424916	+				e	U	2 ± 2	4	4 ± 4	3	0.594	BP4
E111K	not listed				+	f	UD	15 ± 19	5	3 ± 3	3	0.677	
K117R*	VCV000048063	+				e	UD	1 ± 1	3	5 ± 3	3	0.446	BP4
G125A	not listed		+			f	UD	5 ± 4	8	1 ± 1	3	0.385	**neg control** / BP4
G125S	VCV000066894	+				f	U	0 ± 0	3	5 ± 7	3	0.235	BP4
A130P	not listed		+			d	UD	75 ± 4	3	86 ± 15	4	0.868	PS3/PP3
A132P	VCV000066895	+				f	UD	58 ± 26	4	65 ± 31	4	0.746	PS3/BP4
R133L	VCV000014488	+		+	+	g	P	3 ± 5	3	3 ± 5	4	0.811	
R133P	VCV000014508		+			g	LP	77 ± 17	3	81 ± 24	3	0.936	
D136H	VCV000066896				+	c	UD	0 ± 0	3	3 ± 3	3	0.838	
E138K	VCV000066897				+	e	P	2 ± 2	3	3 ± 3	4	0.915	
L140P	VCV000066898		+			g	UD	22 ± 8	3	67 ± 26	4	0.874	PS3/PP3
L140R	VCV000014507				+	g	P	1 ± 1	3	4 ± 6	3	0.702	
S143F	VCV000014510	+			+	c	P	4 ± 3	4	2 ± 1	3	0.83	
S143P	VCV000066899	+				c	P/LP	37 ± 18	5	62 ± 27	4	0.802	
E145K	VCV000014502				+	e	P	5 ± 8	3	2 ± 3	3	0.759	
T150P	VCV000048067		+			c	P/LP	71 ± 9	3	77 ± 22	4	0.759	
E159K	VCV000066900				+	e	UD	3 ± 3	3	4 ± 5	4	0.773	
E161K	VCV000014504	+				g	P/LP	25 ± 21	3	7 ± 9	4	0.739	
L162P	VCV000048068		+			a	LP	63 ± 28	3	82 ± 8	3	0.855	
R166P	VCV000066901	+				e	P/LP	65 ± 11	3	58 ± 19	4	0.808	
R166Q	VCV000163866	+				e	U	3 ± 1	3	1 ± 1	3	0.432	**neg control** / BP4
R166W	VCV000574040					e	U	3 ± 1	3	1 ± 1	3	0.69	**neg control**
L183P	VCV000066904	+				a	UD	79 ± 21	3	75 ± 17	3	0.961	PS3/PP3
R189P	VCV000066907		+			g	UD	52 ± 17	3	63 ± 15	4	0.782	PS3/PP3
R189Q	VCV000392479					g	U	1 ± 1	5	1 ± 1	4	0.456	BP4
R189W	VCV000066906	+				g	U	3 ± 1	3	1 ± 1	4	0.68	**neg control** / BP4
R190Q	VCV000066910	+				a	P/LP	14 ± 8	3	7 ± 1	3	0.925	
D192G	VCV000066911	+				c	U	33 ± 13	4	36 ± 4	3	0.922	PS3/PP3
D192V	VCV000066912	+		+		c	UD	0 ± 0	3	1 ± 1	3	0.914	PP3
N195D	not listed		+			f	UD	46 ± 18	4	62 ± 17	3	0.832	PS3/PP3
N195K	VCV000014483	+				f	P	57 ± 10	6	52 ± 14	3	0.689	
E203K	VCV000048070	+				g	P/LP	7 ± 3	3	6 ± 6	3	0.872	
E203V	VCV000066915	+				g	UD	7 ± 6	4	2 ± 3	3	0.961	PP3
E203G	VCV000014484	+				g	P	44 ± 39	4	6 ± 2	3	0.963	
F206L	VCV000066916		+			c	LP	51 ± 3	4	64 ± 23	3	0.894	
I210S	VCV000048072	+				g	U	45 ± 4	5	61 ± 16	3	0.855	PS3/PP3

Supplementary Table 2. Properties of Coil 1B variants. Variants studied are color-coded by disease-association with cardiac muscle disease (blue), skeletal muscle disease (red), lipodystrophy (yellow), premature aging (gray), or more than one (e.g. R133L) as listed in the UMD-LMNA database. Accession numbers from ClinVar. R166W was not disease-associated and used as negative aggregation control. Location indicates residue position on CCD (see helical wheel diagram in Fig. 1). ClinVar abbreviations designate classifications as of July 2021: pathogenic (P), likely pathogenic (LP), uncertain (U), undetermined (not provided or conflicting interpretation) (UD). HEK 293 and C2C12 myoblast columns lists % of cells that aggregate (mean ± SD) and number of transfections analyzed in N column. Yellow highlight indicates statistical significance determined by ANOVA-Dunn's post hoc test ($P < 0.05$). REVEL column shows scores for each variant filled blue if above the 0.76 threshold (see Supplementary Figure 7) or green if below. Function/Prediction column lists ACMG supporting classifiers for myopathic variants based on our results that are listed as uncertain or undetermined in ClinVar (shaded gray). PS3 represents pathogenic supporting (functional data) if both HEK 293 cells and C2C12 myoblasts show increased aggregation. PP3 and BP4 represent pathongenic or benign supporting (computational data) if REVEL score is above or below the threshold, respectively. Negative control variants (bold) were chosen from being present in genomAD control populations (see Supplementary Table 7). R166W is not disease associated and also used as a negative control. Asterisk: ClinVar shows nearly all possible conditions for K117R and many others we did not study, which are likely incorrect (see also Supplementary Table 4).

Coil 2A	Accession	CM	SM	Lipo	PA	Location	ClinVar	HEK ± SD	N	C2C12 ± SD	N	REVEL	Function / Prediction
WT		-	-	-	-	-	-	3 ± 3	57	2 ± 3	31	-	
L248P	VCV000066930		+			d	UD	62 ± 2	3	76 ± 20	4	0.907	PS3/PP3
R249Q	VCV000066931		+			e	P/LP	34 ± 8	3	76 ± 14	4	0.919	
R249W	VCV000014524		+			e	P/LP	50 ± 17	5	27 ± 42	3	0.903	
Y259H	VCV000066933	+				d	UD	31 ± 10	3	66 ± 27	4	0.905	PS3/PP3
Y259D	VCV000066934		+			d	UD	42 ± 7	3	80 ± 19	3	0.856	PS3/PP3
K260E	not listed		+			e	UD	48 ± 5	3	82 ± 18	3	0.9	PS3/PP3
K260N	not listed	+				e	UD	39 ± 7	3	65 ± 1	3	0.778	PS3/PP3
E262K	not listed				+	g	UD	3 ± 2	3	1 ± 2	3	0.922	
L263P	VCV000066936		+			h	UD	54 ± 6	3	65 ± 25	3	0.923	PS3/PP3
Y267C	VCV000066937		+			a	UD	25 ± 9	3	72 ± 26	3	0.937	PS3/PP3
Y267H	VCV000048084	+	+			a	P/LP	38 ± 16	3	73 ± 8	3	0.929	
S268P	VCV000066938		+			b	UD	39 ± 35	4	81 ± 16	3	0.632	PS3/BP4

Supplementary Table 3. Properties of Coil 2A variants. Variants studied are color-coded by disease-association with cardiac muscle disease (blue), skeletal muscle disease (red), premature aging (gray), or more than one (e.g. Y267H) as listed in the UMD-LMNA database. Accession numbers from ClinVar. Location indicates residue position on CCD (see helical wheel diagram in Fig. 1). ClinVar abbreviations designate classifications as of July 2021: pathogenic (P), likely pathogenic (LP), undetermined (not provided) (UD). HEK 293 and C2C12 myoblast columns lists % of cells that aggregate (mean ± SD) and number of transfections analyzed in N column. Yellow highlight indicates statistical significance determined by ANOVA-Dunn's post hoc test (P < 0.05). REVEL column shows scores for each variant filled blue if above the 0.76 threshold (see Supplementary Figure 7) or green if below. Function/Prediction column lists ACMG supporting classifiers for myopathic variants based on our results that are listed as uncertain or undetermined in ClinVar (shaded gray). PS3 represents pathogenic supporting (functional data) if both HEK and C2C12 cells show increased aggregation. PP3 and BP4 represent pathongenic or benign supporting (computational data) if REVEL score is above or below the threshold, respectively.

Coil 2B	Accession	CM	SM	Lipo	PA	Location	ClinVar	HEK ± SD	N	C2C12 ± SD	N	REVEL	Function / Prediction
WT		-	-	-	-		-	3 ± 3	57	2 ± 3	31		
L292P	not listed		+			a	UD	66 ± 20	3	84 ± 9	3	0.89	PS3/PP3
Q294P	VCV000066949		+			c	UD	48 ± 28	3	72 ± 22	3	0.879	PS3/PP3
S295P	VCV000066950		+			d	UD	51 ± 32	3	71 ± 19	3	0.775	PS3/PP3
S295L	VCV000518983	+				d	U	1 ± 1	3	1 ± 1	3	0.438	**neg control** / BP4
R298C*	VCV000014498	+	+			g	UD	25 ± 24	6	1 ± 1	3	0.876	PP3
I299V	VCV000048092	+		+		a	UD	2 ± 2	3	2 ± 3	3	0.454	**neg control** / BP4
D300G	VCV000217451				+	b	P	4 ± 2	4	1 ± 1	3	0.833	
L302P	VCV000066953		+			d	UD	59 ± 18	4	71 ± 16	4	0.936	PS3/PP3
Q312H	not listed	+	+			g	UD	1 ± 1	3	1 ± 1	3	0.931	PP3
E317K	VCV000048093	+				e	P/LP	7 ± 5	3	1 ± 1	3	0.873	
A318T	VCV000066961	+				f	UD	4 ± 5	3	1 ± 1	3	0.594	**neg control** / BP4
S326T	VCV000048097	+				g	UD	7 ± 5	4	1 ± 2	3	0.484	**neg control** / BP4
R329S*	VCV000292838	+	+	+	+	f	U	4 ± 2	3	0 ± 0	3	0.559	**neg control** / BP4
R331Q	VCV000048098	+	+			a	UD	2 ± 1	3	0 ± 0	3	0.757	**neg control** / BP4
R331P	VCV000066963		+			a	UD	32 ± 14	3	62 ± 20	3	0.858	PS3/PP3
R335Q	VCV000246074	+	+			e	U	3 ± 4	3	1 ± 1	3	0.626	**neg control** / BP4
R335W	VCV000036473	+				e	P/LP	4 ± 1	3	1 ± 2	3	0.819	
R336Q	VCV000066758		+			f	U	9 ± 4	4	1 ± 1	3	0.657	**neg control** / BP4
R343Q	VCV000066759		+			f	U	2 ± 2	3	2 ± 2	3	0.492	**neg control** / BP4
E347K	VCV000066760	+				c	U	4 ± 3	3	0 ± 0	3	0.652	BP4
M348I	VCV000155896	+	+			d	U	10 ± 7	6	2 ± 2	3	0.251	BP4
R349W	VCV000066762	+	+	+		e	P/LP	5 ± 2	3	8 ± 11	3	0.794	
R349L	VCV000066763	+				e	LP	8 ± 3	4	58 ± 49	3	0.871	
A350P	VCV000066764	+				f	UD	38 ± 11	3	6 ± 6	5	0.671	BP4
Q353K	VCV000066766	+				b	UD	41 ± 23	3	47 ± 17	4	0.824	PS3/PP3
Q353R	not listed		+			b	UD	29 ± 18	4	8 ± 14	5	0.878	PP3
D357H	VCV000066769	+				f	U	48 ± 29	5	45 ± 19	4	0.775	PS3/PP3
D357A	not listed	+				f	UD	51 ± 9	4	38 ± 6	3	0.775	PS3/PP3
E358K	VCV000014525		+			g	P	29 ± 20	4	55 ± 6	3	0.953	
E361K	VCV000066772		+			c	U	37 ± 18	3	40 ± 13	4	0.827	PS3/PP3
M371K	VCV000066776		+			f	UD	50 ± 22	3	61 ± 4	3	0.954	PS3/PP3
R377C	VCV000048031	+	+			e	P/LP	28 ± 24	6	2 ± 2	4	0.938	
R377H	VCV000014495	+	+			e	P	39 ± 21	4	9 ± 5	4	0.977	
R377L	VCV000066778	+	+			e	U	62 ± 28	7	67 ± 8	4	0.971	PS3/PP3
L379F	not listed		+			g	UD	49 ± 35	3	56 ± 22	3	0.92	PS3/PP3
L380S	VCV000014523		+			a	P	61 ± 26	3	51 ± 13	3	0.988	
E381A	VCV000066779		+			b	UD	48 ± 27	3	27 ± 15	3	0.938	PS3/PP3
G382R	not listed		+			c	UD	15 ± 6	5	20 ± 21	5	0.946	PS3/PP3
G382V	not listed	+				c	UD	1 ± 2	3	1 ± 2	4	0.972	PP3

Supplementary Table 4. Properties of Coil 2B variants. Variants studied are color-coded by disease-association with cardiac muscle disease (blue), skeletal muscle disease (red), lipodystrophy (yellow), premature aging (gray), or more than one (e.g. R298C) as listed in the UMD-LMNA database. Accession numbers from ClinVar. Asterisk indicates an additional rare linkage to Charcot-Marie Tooth Disease (R298C)[7]. Location indicates residue position on CCD (see helical wheel diagram in Fig. 1). ClinVar abbreviations designate classifications as of July 2021: pathogenic (P), likely pathogenic (LP), uncertain (U), undetermined (not provided or **conflicting interpretation**) (UD). HEK 293 and C2C12 myoblast columns lists % of cells that aggregate (mean ± SD) and number of transfections analyzed in N column. Yellow highlight indicates statistical significance determined by ANOVA-Dunn's post hoc test ($P < 0.05$). REVEL column shows scores for each variant filled blue if above the 0.76 threshold (see Supplementary Figure 7) or green if below. Function/Prediction column lists ACMG supporting classifiers for myopathic variants that are listed as uncertain or undetermined in ClinVar (shaded gray) based on our results. PS3 represents pathogenic supporting (functional data) if both HEK 293 cells and C2C12 myoblasts show increased aggregation. PP3 and BP4 represent pathogenic or benign supporting (computational data) if REVEL score is above or below the threshold, respectively. Negative control variants (bold) were chosen from being present in genomAD control populations (see Supplementary Table 7). Asterisk: ClinVar shows nearly all possible conditions for R329S (not listed in UMD), which is likely incorrect. ClinVar also reports the same condtions for R335W and R349W not in agreement with the UMD.

IgD	Accession	CM	SM	Lipo	PA	Location	ClinVar	HEK ± SD	N	C2C12 ± SD	N	DB Sol	N	FoldX	REVEL	Function / Prediction
WT		-	-	-	-	-	-	3 ± 3	57	2 ± 3	31	100 ± 6	5	-	-	
R435C	VCV000066802	+			+	0.141	U	6 ± 4	6	4 ± 5	4	100 ± 24	3	-0.15	0.667	BP4
R439C	VCV000066804			+		0.257	U	10 ± 11	6	3 ± 2	4	98 ± 17	3	0.71	0.649	
V440M	VCV000014520	+		+		0.122	U	15 ± 15	7	1 ± 2	3	92 ± 1	2	4.22	0.926	PP3
A441V	not listed	+				0.123	UD	6 ± 6	5	2 ± 2	4	93 ± 9	3	-0.42	0.612	BP4
V442A	not listed		+			n/a	UD	28 ± 14	5	3 ± 4	4	9 ± 7	3	n/a	0.954	PP3
V445E	not listed	+				n/a	UD	22 ± 3	5	2 ± 3	4	112 ± 22	3	n/a	0.943	PP3
D446V	VCV000066805		+			0.103	U	28 ± 24	5	3 ± 4	4	0 ± 0	3	3.49	0.979	PP3
G449D	VCV000066806		+			0.083	P	21 ± 13	8	6 ± 6	4	70 ± 1	3	10.35	0.975	
G449V	not listed		+			0.083	UD	27 ± 20	8	8 ± 8	6	0 ± 0	4	11.06	0.981	PS3/PP3
R453P	VCV000066808		+			0.143	UD	35 ± 21	5	27 ± 9	4	20 ± 15	3	5.71	0.957	PS3/PP3
R453W	VCV000014478		+	+		0.143	P/LP	32 ± 14	6	3 ± 5	4	6 ± 6	3	9.03	0.881	
L454P	VCV000066809		+			0.119	UD	49 ± 16	6	48 ± 20	3	3 ± 5	3	6.04	0.987	PS3/PP3
R455P	VCV000066810		+			0.383	UD	47 ± 13	5	33 ± 22	4	4 ± 7	3	4.07	0.834	PS3/PP3
N456D	VCV000066811		+			0.072	P	36 ± 12	5	4 ± 4	3	49 ± 23	3	2.81	0.937	
N456H	not listed		+			0.072	UD	30 ± 17	5	2 ± 2	3	11 ± 12	3	10.8	0.948	PS3/PP3
N456I	VCV000066812		+			0.072	UD	44 ± 19	7	35 ± 9	4	1 ± 3	4	1.38	0.941	PS3/PP3
N456K	VCV000842074		+			0.072	P	35 ± 17	4	43 ± 16	3	5 ± 8	3	10.52	0.926	
N459S	VCV000178062		+			0.741	UD	2 ± 2	4	4 ± 6	3	103 ± 20	3	0.12	0.394	BP4
D461Y	VCV000066819	+				0.282	U	8 ± 6	5	4 ± 6	4	54 ± 39	3	1.14	0.933	PP3
G465D	VCV000014493			+		0.129	P	6 ± 5	5	7 ± 6	4	105 ± 30	3	1.08	0.931	
W467R	VCV000066821		+			0.1	LP	49 ± 16	6	19 ± 17	3	1 ± 2	3	4.29	0.959	
I469T	VCV000066823		+			0.083	UD	31 ± 15	4	2 ± 2	4	16 ± 10	3	3.52	0.943	PP3
R471C	VCV000014503			+		0.08	U	7 ± 5	4	n/a		n/a		n/a	0.901	
R471G	VCV000066824		+	+		0.08	UD	10 ± 8	5	4 ± 7	3	75 ± 7	3	5.86	0.915	PP3
R471H	VCV000036476	+	+			0.08	P/LP	16 ± 27	6	3 ± 3	3	n/a		n/a	0.898	
Y481H	VCV000066826		+			0.078	UD	4 ± 4	4	5 ± 1	3	9 ± 9	3	1.8	0.982	PP3
R482W	VCV000014489		+	+		0.475	P	23 ± 26	7	7 ± 7	3	83 ± 14	3	-0.4	0.837	
P485R	not listed			+		0.517	UD	3 ± 4	5	0 ± 0	3	30 ± 17	3	2.45	0.572	
K486N	VCV000066829			+		0.638	P	2 ± 2	4	1 ± 2	3	59 ± 29	3	0.54	0.639	
T488P	VCV000066830	+				0.265	UD	6 ± 7	4	7 ± 2	3	54 ± 7	3	0.98	0.833	PP3
L489P	not listed		+			0.069	UD	34 ± 19	7	18 ± 23	3	6 ± 9	4	6.17	0.965	PS3/PP3
W498C	VCV000066838	+	+			0.218	LP	17 ± 15	9	1 ± 1	2	16 ± 10	3	4.32	0.954	PP3
W498R	VCV000066836		+			0.218	UD	11 ± 8	6	2 ± 2	3	9 ± 2	3	4.16	0.897	PP3
L512P	VCV000066844		+			0.124	U	25 ± 15	4	4 ± 1	3	0 ± 0	3	5.26	0.958	PP3
W514R	VCV000246077		+			0.096	P	13 ± 10	10	3 ± 4	3	8 ± 7	4	4.83	0.96	
W520G	VCV000066845		+			0.118	UD	12 ± 11	5	3 ± 2	3	17 ± 4	3	7.29	0.978	PP3
W520R	VCV000476824		+			0.118	P	22 ± 15	4	3 ± 3	3	1 ± 2	3	n/a	0.984	
W520S	VCV000066846		+			0.118	UD	24 ± 9	5	2 ± 2	3	3 ± 5	3	7.25	0.94	PP3
G523R	VCV000200946	+				0.377	U	10 ± 11	5	0 ± 0	3	33 ± 7	3	2.8	0.957	PP3
R527C	VCV000014487				+	0.237	U	2 ± 2	5	5 ± 3	3	156 ± 41	3	-0.1	0.862	
R527H	VCV000014499			+	+	0.237	U	4 ± 3	4	2 ± 2	3	n/a		n/a	0.734	
R527P	VCV000014481		+	+		0.237	P	22 ± 12	4	4 ± 2	3	91 ± 9	3	3.07	0.884	
T528K	VCV000066849		+		+	0.128	P/LP	34 ± 9	5	2 ± 2	3	0 ± 0	3	7.11	0.923	
T528M	VCV000066851			+	+	0.128	U	8 ± 11	5	4 ± 3	3	80 ± 9	3	2.98	0.889	
T528R	VCV000066850		+	+	+	0.128	P/LP	26 ± 6	4	4 ± 3	3	5 ± 2	3	10.29	0.927	
A529T	VCV000014522				+	0.1	P	5 ± 6	4	3 ± 2	3	163 ± 13	3	0.38	0.488	
A529V	VCV000014513				+	0.1	P	4 ± 3	5	4 ± 2	3	139 ± 27	4	-1.14	0.492	
L530P	VCV000014482		+			0.094	P	17 ± 17	5	3 ± 3	3	14 ± 25	3	7.58	0.982	
M540T	VCV000066858				+	0.067	P	7 ± 7	5	4 ± 2	3	158 ± 50	3	0.71	0.62	
R541C	VCV000048046	+				0.148	P/LP	10 ± 13	6	3 ± 3	3	122 ± 40	3	-0.17	0.887	
R541G	VCV000029775	+				0.148	P	33 ± 8	4	4 ± 5	3	115 ± 47	3	1.26	0.864	
R541H	VCV000066860	+	+			0.148	P/LP	9 ± 7	7	3 ± 4	3	124 ± 29	3	-0.95	0.905	
R541S	VCV000066859	+	+			0.148	LP	16 ± 12	6	3 ± 2	3	89 ± 31	3	-0.27	0.882	
R541P	VCV000066861	+	+			0.148	P/LP	16 ± 14	6	4 ± 2	3	80 ± 27	3	6.17	0.975	
K542N	VCV000014509				+	0.274	P	8 ± 3	4	3 ± 3	3	174 ± 38	3	0.9	0.605	
R545C	VCV000066862	+	+			n/a	U	5 ± 6	7	5 ± 3	3	115 ± 24	3	n/a	0.828	PP3*

Supplementary Table 5. Properties of IgD variants. Variants studied are color-coded by disease-association with cardiac muscle disease (blue), skeletal muscle disease (red), lipodystrophy (yellow), premature aging (gray), or more than one (e.g. R435C) as listed in the UMD-LMNA database. Accession numbers from ClinVar. Location indicates solvent accessible surface area for each IgD variant (the lower the more buried) from Scharner et al 2013 based on crystal structure PDB: 1IFR (see helical wheel diagram in Fig. 1). ClinVar abbreviations designate classifications as of July 2021: pathogenic (P), likely pathogenic (LP), uncertain (U), undetermined (not provided or **conflicting interpretation**) (UD). HEK 293 and C2C12 columns lists % of cells that aggregate (mean ± SD) and number of transfections analyzed (N). Yellow highlight indicates statistical significance determined by ANOVA-Dunn's post hoc test ($P < 0.05$). Dot Blot Solubility (DB Sol) column lists % recombinant PASD solubility compared to WT (mean ± standard deviation) and number of experiments (N). REVEL column shows scores for each variant filled blue if above the 0.76 threshold (see Supplementary Figure 7) or green if below. Function/Prediction column lists ACMG supporting classifiers for myopathic variants based on our results that are listed as uncertain or undetermined in ClinVar (shaded gray). PS3 represents pathogenic supporting (functional data) if both HEK 293 cells and C2C12 myoblasts show increased aggregation. PP3 and BP4 represent pathogenic or benign supporting (computational data) if REVEL score is above or below the threshold, respectively. Negative Control variants (bold) were determined from being present in genomAD control populations (see Supplementary Table 7). Asterisk: R545C is an example of a skeletal muscle laminopathy that does not aggregate but shows other abnormalities in patient myocytes.

LMNA	iPSC-CM ± STD	N
WT	1 ± 1	5
L35P	82 ± 7	3
S143P	74 ± 6	3
S143F	1 ± 1	3
L183P	75 ± 14	3
D192G	58 ± 27	3
D192V	1 ± 1	3
N195K	61 ± 10	3
I210S	73 ± 9	3
Q353K	84 ± 5	3
R377L	89 ± 4	3

Supplementary Table 6. Aggregation properties of variants in iPSC-CMs. Variants studied are color-coded by disease-association with cardiac muscle disease (blue), skeletal muscle disease (red), lipodystrophy (yellow), premature aging (gray), or more than one (e.g. D192V) as listed in the UMD-LMNA database. iPSC-CM column lists % of cells that aggregate (mean ± SD) and number of transfections analyzed in N column. Yellow highlight indicates statistical significance determined by Tukey's post hoc test ($P < 0.05$).

Variant	gnomAD total	gnomAD controls	ExAC	ClinVar Variant Studied
R50H	2	1	0	R50H
R101H	1	0	0	R101P
**R110H**	13	7	6	R110H
R110S	1	0	0	R110S
K117R	16	0	6	K117R
**G125A**	2	2	0	G125A
A132S	2	0	2	A132P
R133G	3	0	0	R133L
E159Q	1	0	0	E159K
**R166Q/W**	10/7	6/5	2/2	R166Q / R166W
L183F	1	1	1	L183P
**R189**Q/**W**	1/5	1/3	1/2	R189P / R189Q / R189W
L263M/V	1/1	0/1	0	L263P
S295L	7	0	4	S295L / S295P
R298C	9	2	5	R298C
**I299V**/M	123/2	55/0	47/0	I299V
E317D	1	1	0	E317K
**A318T**/V	5/2	2/0	0	A318T
**S326T**/L	15/4	11/1	7/0	S326T
**R329**C/G/H/**S**	1/2/5/6	1/0/3/4	0/0/0/2	R329S
R331E/Q/W	0/3/3	0/2/1	0/2/0	R331Q
**R335Q**	20	13	4	R335Q
**R336Q**	7	3	1	R336Q
**R343Q**/W	10/1	6/0	8/0	R343Q
R349Q	1	0	0	R349W
A350T	1	0	0	A350P
M371V	1	1	0	M371K
G382D	1	0	0	G382R
R435C	2	1	1	R435C
R439C	8	2	0	R439C
V442M	10	4	4	V442A
D446E	1	1	0	D446V
R453Q	3	2	3	R453P
R455C	2	0	0	R455P
**N459S**	7	3	2	N459S
D461E/Y	1/2	1/1	0/1	D461Y
R471C	3	2	3	R471C / R471G / R471H
R482W	1	0	0	R482W
**G523R**	22	12	8	G523R
R527C/H	5/10	3/4	0/4	R527C / R527H
T528M	2	2	0	T528M
A529T	1	0	0	A529T
L530V	2	0	1	L530P
K542R	2	0	0	K542N
R545C/H	0/48	0/13	4/0	R545C

Supplementary Table 7. Variant allele frequency in general population. Variants listed in the Genome Aggregation Database (genomAD) v2.1.1 and Exome Aggregation Consortium (ExAC) found at https://gnomad.broadinstitute.org that we characterized herein. gnomAD (total) column numbers are the total allele counts in 141,456 genome/exome sequences and gnomAD (control) column numbers are the control allele counts in 60,146 exome/genome sequences. ClinVar variants characterized herein are color-coded by disease-association with cardiac muscle disease (blue), skeletal muscle disease (red), lipodystrophy (yellow), premature aging (gray), or more than one (e.g. R101P) as listed in the UMD-LMNA database. Variants in bold were chosen as negative controls for being in the gnomAD control population. Asterisk indicates negative control variant with no disease linkage.

Variants	Forward Primers	Reverse Primers
E33D	ctgcaggagaaggatgacctgcaggagct	agctcctgcaggtcatccttctcctgcag
E33G	ctgcaggagaagggggacctgcaggag	ctcctgcaggtcccccttctcctgcag
L35V	caggagaaggaggacgtgcaggagctcaatg	cattgagctcctgcacgtcctccttctcctg
L35P	ggagaaggaggacccgcaggagctcaatg	cattgagctcctgcgggtcctccttctcc
N39D	acctgcaggagctcgatgatcgcttggcg	cgccaagcgatcatcgagctcctgcaggt
N39S	gacctgcaggagctcagtgatcgcttggcg	cgccaagcgatcactgagctcctgcaggtc
N39Y	acctgcaggagctctatgatcgcttggcg	cgccaagcgatcatagagctcctgcaggt
R41C	acctgcaggagctcaatgattgcttggcggtc	gaccgccaagcaatcattgagctcctgcaggt
R41S	acctgcaggagctcaatgatagcttggcggtc	gaccgccaagctatcattgagctcctgcaggt
A43T	gctcaatgatcgcttgacggtctacatcgaccg	cggtcgatgtagaccgtcaagcgatcattgagc
Y45C	cgcttggcggtctgcatcgaccgtgtg	cacacggtcgatgcagaccgccaagcg
I46V	cttggcggtctacgtcgaccgtgtgcg	cgcacacggtcgacgtagaccgccaag
D47H	ggcggtctacatccaccgtgtgcgctc	gagcgcacacggtggatgtagaccgcc
R50H	catcgaccgtgtgcactcgctggaaacgg	ccgtttccagcgagtgcacacggtcgatg
R50P	atcgaccgtgtgccctcgctggaaacg	cgtttccagcgagggcacacggtcgat
R50S	acatcgaccgtgtgagctcgctggaaacg	cgtttccagcgagctcacacggtcgatgt
L52P	cgtgtgcgctcgccggaaacggagaac	gttctccgtttccggcgagcgcacacg
E53V	gtgcgctcgctggtaacggagaacgca	tgcgttctccgttaccagcgagcgcac
A57P	gaaacggagaacccagggctgcgcc	ggcgcagccctgggttctccgtttc
L59R	gagaacgcagggcggcgccttcgcatc	gatgcgaaggcgccgccctgcgttctc
R60G	gaacgcagggctgggccttcgcatcac	gtgatgcgaaggcccagccctgcgttc
I63S	gctgcgccttcgcagcaccgagtctgaag	cttcagactcggtgctgcgaaggcgcagc
I63N	gctgcgccttcgcaacaccgagtctgaag	cttcagactcggtgttgcgaaggcgcagc
E65G	ccttcgcatcaccgggtctgaagaggtgg	ccacctcttcagacccggtgatgcgaagg
E82K	aaggccgcctacaaggccgagctcg	cgagctcggccttgtaggcggcctt
L85P	cgaggccgagcccggggatgccc	gggcatccccgggctcggcctcg
L85R	cgaggccgagcgcggggatgccc	gggcatccccgcgctcggcctcg
R89C	gctcggggatgcctgcaagacccttga	tcaagggtcttgcaggcatccccgagc
R89L	ctcggggatgccctcaagacccttgac	gtcaagggtcttgagggcatccccgag
L92F	gatgcccgcaagacctttgactcagtagcca	tggctactgagtcaaaggtcttgcgggcatc
K97E	gactcagtagccgaggagcgcgccc	gggcgcgctcctcggctactgagtc
R101P	ggagcgcgccccccctgcagctgg	ccagctgcagggggggcgcgctcc
L102Q	gcgcgcccgccagcagctggagc	gctccagctgctggcgggcgcgc
R110H	ggagctgagcaaagtgcatgaggagtttaaggagc	gctccttaaactcctcatgcactttgctcagctcc
R110S	tggagctgagcaaagtgagtgaggagtttaaggag	ctccttaaactcctcactcactttgctcagctcca
E111K	ctggagctgagcaaagtgcgtaaggagtttaagg	ccttaaactccttacgcactttgctcagctccag
K117R	gcgtgaggagtttaaggagctgagagcgcgcaatac	gtattgcgcgctctcagctccttaaactcctcacgc
G125A	caataccaagaaggaggctgacctgatagctgctc	gagcagctatcaggtcagcctccttcttggtattg
G125S	gcaataccaagaaggagagtgacctgatagctgct	agcagctatcaggtcactctccttcttggtattgc
A130P	gtgacctgatagctcctcaggctcggctg	cagccgagcctgaggagctatcaggtcac
A132P	tgatagctgctcagcctcggctgaaggac	gtccttcagccgaggctgagcagctatca
R133L	gctgctcaggctctgctgaaggacctg	caggtccttcagcagagcctgagcagc

R133P	gctgctcaggctccgctgaaggacctg	caggtccttcagcggagcctgagcagc
D136H	gctcggctgaagcacctggaggctc	gagcctccaggtgcttcagccgagc
E138K	ggctgaaggacctgaaggctctgctgaac	gttcagcagagccttcaggtccttcagcc
L140P	ggacctggaggctccgctgaactccaagg	ccttggagttcagcggagcctccaggtcc
L140R	ggacctggaggctcggctgaactccaagg	ccttggagttcagccgagcctccaggtcc
S143F	ggctctgctgaacttcaaggaggccgcac	gtgcggcctccttgaagttcagcagagcc
S143P	ggctctgctgaaccccaaggaggccgc	gcggcctccttggggttcagcagagcc
E145K	tgctgaactccaagaaggccgcactgagc	gctcagtgcggccttcttggagttcagca
T150P	ggccgcactgagccctgctctcagtga	tcactgagagcagggctcagtgcggcc
E159K	aagcgcacgctgaagggcgagctgc	gcagctcgcccttcagcgtgcgctt
E161K	gcacgctggagggcaagctgcatgatctg	cagatcatgcagcttgccctccagcgtgc
L162P	ctggagggcgagccgcatgatctgcgg	ccgcagatcatgcggctcgccctccag
R166P	tgcatgatctgccgggccaggtggc	gccacctggcccggcagatcatgca
R166Q	ctgcatgatctgcagggccaggtggcc	ggccacctggccctgcagatcatgcag
R166W	gctgcatgatctgtggggccaggtggc	gccacctggccccacagatcatgcagc
L183P	gaggccaagaagcaacctcaggatgagatgctg	cagcatctcatcctgaggttgcttcttggcctc
R189P	ggatgagatgctgtggcgggtggatgc	gcatccacccgccacagcatctcatcc
R189Q	cagcatccacccgctgcagcatctcatcc	ggatgagatgctgcagcgggtggatgctg
R189W	ggatgagatgctgtggcgggtggatgc	gcatccacccgccacagcatctcatcc
R190Q	gagatgctgcggcaggtggatgctgag	ctcagcatccacctgccgcagcatctc
D192G	ctgcggcgggtgggtgctgagaacagg	cctgttctcagcacccacccgccgcag
D192V	ctgcggcgggtggttgctgagaacagg	cctgttctcagcaaccacccgccgcag
N195D	gggtggatgctgaggacaggctgcagacc	ggtctgcagcctgtcctcagcatccaccc
N195K	cgggtggatgctgagaagaggctgcaga	tctgcagcctcttctcagcatccacccg
E203K	tgcagaccatgaaggagaaactggacttccagaag	cttctggaagtccagtttctccttcatggtctgca
E203V	cagaccatgaaggaggtactggacttccagaag	cttctggaagtccagtacctccttcatggtctg
E203G	cagaccatgaaggagggactggacttccagaag	cttctggaagtccagtccctccttcatggtctg
F206L	atgaaggaggaactggacttacagaagaacatctacag	ctgtagatgttcttctgtaagtccagttcctccttcat
I210S	ggacttccagaagaacagctacagtgaggagctgc	gcagctcctcactgtagctgttcttctggaagtcc
L248P	gctgcaggaaccgcgggcccagc	gctgggcccgcggttcctgcagc
R249Q	ctgcaggaactgcaggcccagcatgag	ctcatgctgggcctgcagttcctgcag
R249W	gctgcaggaactgtgggcccagcatga	tcatgctgggcccacagttcctgcagc
Y259H	gaccaggtggagcagcataagaaggagctgg	ccagctccttcttatgctgctccacctggtc
Y259D	gaccaggtggagcaggataagaaggagctgg	ccagctccttcttatcctgctccacctggtc
K260E	gaggaccaggtggagcagtatgagaaggagctg	cagctccttctcatactgctccacctggtcctc
K260N	aggaccaggtggagcagtataataaggagctggag	ctccagctccttattatactgctccacctggtcct
E262K	ggtggagcagtataagaagaagctggagaagacttattc	gaataagtcttctccagcttcttcttatactgctccacc
L263P	agcagtataagaaggagccggagaagacttattctgc	gcagaataagtcttctccggctccttcttatactgct
Y267C	aagaaggagctggagaagacttgttctgccaagct	agcttggcagaacaagtcttctccagctccttctt
Y267H	ggagctggagaagactcattctgccaagctgga-	tccagcttggcagaatgagtcttctccagctcc
S268P	gaaggagctggagaagacttatcctgccaagctgg	ccagcttggcaggataagtcttctccagctccttc
L292P	cccacgaggagccgcagcagtcgcg	cgcgactgctgcggctcctcgtggg
Q294P	aggagctgcagccgtcgcgcatccg	cggatgcgcgacggctgcagctcct

S295P	gagctgcagcagccgcgcatccgca	tgcggatgcgcggctgctgcagctc
S295L	cgaggagctgcagcagttgcgcatccg	cggatgcgcaactgctgcagctcctcg
R298C	gcagtcgcgcatctgcatcgacagcct	aggctgtcgatgcagatgcgcgactgc
I299V	gtcgcgcatccgcgtcgacagcctctc	gagaggctgtcgacgcggatgcgcgac
D300G	cgcatccgcatcggcagcctctctgcc	ggcagagaggctgccgatgcggatgcg
L302P	cgcatcgacagcccctctgcccagctc	gagctgggcagaggggctgtcgatgcg
Q312H	cagctccagaagcatctggcagccaagga	tccttggctgccagatgcttctggagctg
E317K	gctggcagccaagaaggcgaagcttcg	cgaagcttcgccttcttggctgccagc
A318T	tggcagccaaggagacgaagcttcgagac	gtctcgaagcttcgtctccttggctgcca
S326T	gctcacgggccagcgtgtcctccaggtct	agacctggaggacacgctggcccgtgagc
R329S	ggactcactggccagtgagcgggacac	gtgtcccgctcactggccagtgagtcc
R331Q	ggctggtgtcctgctcacgggcc	ggcccgtgagcaggacaccagcc
R331P	ggcccgtgagccggacaccagcc	ggctggtgtccggctcacgggcc
R335Q	ggacaccagccagcggctgctgg	ccagcagccgctggctggtgtcc
R335W	gggacaccagctggcggctgctg	cagcagccgccagctggtgtccc
R336Q	caccagccggcagctgctggcgg	ccgccagcagctgccggctggtg
R343Q	gcggaaaaggagcaggagatggccgag	ctcggccatctcctgctccttttccgc
E347K	cttgcccgcatcttggccatctcccgc	gcgggagatggccaagatgcgggcaag
M348I	ccttgcccgtatctcggccatctccc-	gggagatggccgagatacgggcaagg
R349W	ctgcatccttgcccacatctcggccatct	agatggccgagatgtgggcaaggatgcag
R349L	gctgcatccttgccagcatctcggccatc	gatggccgagatgctggcaaggatgcagc
A350P	gctgcatccttggccgcatctcggc	gccgagatgcggccaaggatgcagc
Q353K	tccagctgctgcttcatccttgcccgc	gcgggcaaggatgaagcagcagctgga
Q353R	gtccagctgctgccgcatccttgcccg	cgggcaaggatgcggcagcagctggac
D357H	tcctggtactcgtgcagctgctgctgc	gcagcagcagctgcacgagtaccagga
D357A	ctcctggtactcggccagctgctgctg	cagcagcagctggccgagtaccaggag
E358K	gaagctcctggtacttgtccagctgctgctg	cagcagcagctggacaagtaccaggagcttc
E361K	ttgatgtccagaagcttctggtactcgtccagc	gctggacgagtaccagaagcttctggacatcaa
M371K	cgtggatctccttgtccagggccagct	agctggccctggacaaggagatccacg
R377C	gagatccacgcctactgcaagctcttggagg	cctccaagagcttgcagtaggcgtggatctc
R377H	cctccaagagcttgtggtaggcgtggatc	gatccacgcctaccacaagctcttggagg
R377L	cctccaagagcttgaggtaggcgtggatc	gatccacgcctacctcaagctcttggagg
L379F	tcgccctccaagaacttgcggtaggcg	cgcctaccgcaagttcttggagggcga
L380S	tcgccctccgagagcttgcggtaggcg	cgcctaccgcaagctctcggagggcga
E381A	ctcctcctcgcccgccaagagcttgcg	cgcaagctcttggcgggcgaggaggag
G382R	ctctcctcctcgcgctccaagagcttg	caagctcttggagcgcgaggaggagag
G382V	caagctcttggaggtcgaggaggagaggc	gcctctcctcctcgacctccaagagcttg
R435C	gcccgctagtgcatgcgtgctgtgaga	tctcacagcacgcatgcactagcgggc
R439C	cacggccacgcacccgctagtgc	gcactagcgggtgcgtggccgtg
V440M	ctccacggccatgcgcccgctag	ctagcgggcgcatggccgtggag
A441V	cctcctccacgaccacgcgcccg	cgggcgcgtggtcgtggaggagg
V442A	ccacctcctccgcggccacgcgc	gcgcgtggccgcggaggaggtgg
V445E	ccctcctcatcctcctcctccacgg	ccgtggaggaggaggatgaggaggg

D446V	cttgccctcctcaaccacctcctccac	gtggaggaggtggttgaggagggcaag
G449D	gccggacaaacttgtcctcctcatccacc	ggtggatgaggaggacaagtttgtccggc
G449V	gccggacaaacttgacctcctcatccacc	ggtggatgaggaggtcaagtttgtccggc
R453P	acttgttgcgcagcgggacaaacttgccc	gggcaagtttgtcccgctgcgcaacaagt
R453W	ttgttgcgcagccagacaaacttgccctcc	ggagggcaagtttgtctggctgcgcaacaa
L454P	ttggacttgttgcgcggccggacaaacttgc	gcaagtttgtccggccgcgcaacaagtccaa
R455P	ctcattggacttgttgggcagccggacaaactt	aagtttgtccggctgcccaacaagtccaatgag
N456D	cctcattggacttgtcgcgcagccggacaaa	tttgtccggctgcgcgacaagtccaatgagg
N456H	cctcattggacttgtggcgcagccggacaaa	tttgtccggctgcgccacaagtccaatgagg
N456I	cctcattggacttgatgcgcagccggac	gtccggctgcgcatcaagtccaatgagg
N456K	cctcattggacttcttgcgcagccggac	gtccggctgcgcaagaagtccaatgagg
N459S	tggactggtcctcactggacttgttgcgcag	ctgcgcaacaagtccagtgaggaccagtcca
D461Y	ccaattgcccatggactgatactcattggacttgttgcg	cgcaacaagtccaatgagtatcagtccatgggcaattgg
N456D	cctcattggacttgtcgcgcagccggacaaa	tttgtccggctgcgcgacaagtccaatgagg
W467R	gcttgatctgcctattgcccatggactggtc	gaccagtccatgggcaataggcagatcaagc
I469T	ccattctggcgcttggtctgccaattgccca	tgggcaattggcagaccaagcgccagaatgg
R471C	atcatctccattctggcacttgatctgccaattgc	gcaattggcagatcaagtgccagaatggagatgat
R471G	atcatctccattctggcccttgatctgccaattgc	gcaattggcagatcaagggccagaatggagatgat
R471H	gatcatctccattctggtgcttgatctgccaattg	caattggcagatcaagcaccagaatggagatgatc
Y481H	tgggaaccggtgagtcagcaagggatcatctcc	ggagatgatcccttgctgactcaccggttccca
R482W	gaactttggtgggaaccagtaagtcagcaagggat	atcccttgctgacttactggttcccaccaaagttc
P485R	ctgacttaccggttcccaagaaagttcaccctgaaggc	gccttcagggtgaactttcttgggaaccggtaagtcag
K486N	gccttcagggtgaaatttggtgggaaccgg	ccggttcccaccaaatttcaccctgaaggc
T488P	cccagccttcaggggggaactttggtggga	tcccaccaaagttcccccctgaaggctggg
L489P	cctgcccagccttcggggtgaactttggt	accaaagttcaccccgaaggctgggcagg
W498C	cagctcctgcagcgcagatcgtcacca	tggtgacgatctgcgctgcaggagctg
W498R	gctcctgcagccctgatcgtcaccacc	ggtggtgacgatcagggctgcaggagc
L512P	gtgccttccacactaggtcggtagggg	cccctaccgacctagtgtggaaggcac
W514R	gttctgtgccttcctcaccaggtcggtag	ctaccgacctggtgaggaaggcacagaac
W520G	tcccgcagcccccggtgttctgtgc	gcacagaacaccgggggctgcggga
W520R	tcccgcagcccctggtgttctgtgc	gcacagaacaccaggggctgcggga
W520S	ccgcagcccgaggtgttctgtgcctt	aaggcacagaacacctcgggctgcgg
G523R	gcaggctgttcctgcagccccaggt	acctggggctgcaggaacagcctgc
R527C	gatgagagccgtacacaggctgttcccgc	gcgggaacagcctgtgtacggctctcatc
R527H	tgatgagagccgtatgcaggctgttcccg	cgggaacagcctgcatacggctctcatca
R527P	gatgagagccgtaggcaggctgttccc	gggaacagcctgcctacggctctcatc
T528K	gttgatgagagccttacgcaggctgttccc	gggaacagcctgcgtaaggctctcatcaac
T528M	gttgatgagagccatacgcaggctgttccc	gggaacagcctgcgtatggctctcatcaac
T528R	gggaacagcctgcgtagggctctcatcaac	gttgatgagagccctacgcaggctgttccc
A529T	gtggagttgatgagagtcgtacgcaggctgttc	gaacagcctgcgtacgactctcatcaactccac
A529V	gtggagttgatgagaaccgtacgcaggctgt	acagcctgcgtacggttctcatcaactccac
L530P	cagtggagttgatgggagccgtacgcagg	cctgcgtacggctcccatcaactccactg
M540T	cagcttgcgcgtggccacttcttcccc	ggggaagaagtggccacgcgcaagctg

R541C	aagaagtggccatgtgcaagctggtgcgc	gcgcaccagcttgcacatggccacttctt
R541G	agaagtggccatgggcaagctggtgcg	cgcaccagcttgcccatggccacttct
R541H	gaagtggccatgcacaagctggtgcgc	gcgcaccagcttgtgcatggccacttc
R541S	gcgcaccagcttgctcatggccacttctt	aagaagtggccatgagcaagctggtgcgc
R541P	gcgcaccagcttgggcatggccacttc	gaagtggccatgcccaagctggtgcgc
K542N	gagcgcaccagattgcgcatggccactt	aagtggccatgcgcaatctggtgcgctc
R545C	cacagtcactgagcacaccagcttgcgca	tgcgcaagctggtgtgctcagtgactgtg

Supplementary Table 8. Mutagenic primers. Forward and reverse mutagenic primers designed by the Agilent QuikChange Primer Design tool are listed for each variant.
