# Supplementary material for: Most myopathic lamin variants aggregate: a functional genomics approach for assessing variants of uncertain significance
Source: NPJ Genom Med. 2021 Dec 3;6:103. doi: 10.1038/s41525-021-00265-x (PMC8642518; doi:10.1038/s41525-021-00265-x)
Supplement: Supplementary file 2 — Reporting Summary [file 41525_2021_265_MOESM2_ESM.pdf]

nature research

Corresponding author(s): Corey L Anderson and Lee L Eckhardt

Last updated by author(s): October 9, 2021

Reporting Summary

Nature Research wishes to improve the reproducibility of the work that we publish. This form provides structure for consistency and transparency in reporting. For further information on Nature Research policies, see our Editorial Policies and the Editorial Policy Checklist.

Statistics

For all statistical analyses, confirm that the following items are present in the figure legend, table legend, main text, or Methods section.

n/a	Confirmed	
☐	☒	The exact sample size (*n*) for each experimental group/condition, given as a discrete number and unit of measurement
☐	☒	A statement on whether measurements were taken from distinct samples or whether the same sample was measured repeatedly
☐	☒	The statistical test(s) used AND whether they are one- or two-sided<br>*Only common tests should be described solely by name; describe more complex techniques in the Methods section.*
☒	☐	A description of all covariates tested
☒	☐	A description of any assumptions or corrections, such as tests of normality and adjustment for multiple comparisons
☐	☒	A full description of the statistical parameters including central tendency (e.g. means) or other basic estimates (e.g. regression coefficient) AND variation (e.g. standard deviation) or associated estimates of uncertainty (e.g. confidence intervals)
☐	☒	For null hypothesis testing, the test statistic (e.g. *F*, *t*, *r*) with confidence intervals, effect sizes, degrees of freedom and *P* value noted<br>*Give P values as exact values whenever suitable.*
☒	☐	For Bayesian analysis, information on the choice of priors and Markov chain Monte Carlo settings
☒	☐	For hierarchical and complex designs, identification of the appropriate level for tests and full reporting of outcomes
☒	☐	Estimates of effect sizes (e.g. Cohen's *d*, Pearson's *r*), indicating how they were calculated

Our web collection on statistics for biologists contains articles on many of the points above.

Software and code

Policy information about availability of computer code

Data collection	REVEL scores were obtained by entering chromosomal coordinates of variants obtained from ClinVar into the VariED database (varied.cgm.ntu.edu.tw). FoldX values were obtained from a publication referenced.
Data analysis	FlowJo for flow cytometry analysis, ImageJ for immunoblot densitometry analysis, PAST (PAleontological STatistics) software package version 3 for SD and one-way ANOVA analysis, Excel version16.16.27 for figure generation, and Epitools web calculator for ROC-AUC analysis and associated supplementary figures.

For manuscripts utilizing custom algorithms or software that are central to the research but not yet described in published literature, software must be made available to editors and reviewers. We strongly encourage code deposition in a community repository (e.g. GitHub). See the Nature Research guidelines for submitting code & software for further information.

Data

Policy information about availability of data

All manuscripts must include a data availability statement. This statement should provide the following information, where applicable:

- Accession codes, unique identifiers, or web links for publicly available datasets
- A list of figures that have associated raw data
- A description of any restrictions on data availability

All data analyzed in this study is available upon reasonable request. Raw data and ClinVar accession numbers are provided in the Supplementary Tables for all variants where available.

Field-specific reporting

Please select the one below that is the best fit for your research. If you are not sure, read the appropriate sections before making your selection.

☒ Life sciences ☐ Behavioural & social sciences ☐ Ecological, evolutionary & environmental sciences

For a reference copy of the document with all sections, see nature.com/documents/nr-reporting-summary-flat.pdf

Life sciences study design

All studies must disclose on these points even when the disclosure is negative.

Sample size	All sample sizes were n ≥ 3.
Data exclusions	no data was excluded from this manuscript
Replication	Biological triplicates (at a minimum) were performed for WT and all variants analyzed by aggregation or solubility.
Randomization	Most LMNA variants listed in the UMD-LMNA database (most also in the Clinvar database) that fell within each of Lamin A structural domains. were selected. All variants within each domain were analyzed together and compared to WT.
Blinding	All of the images analyzed (i.e. counting cells with aggregates) were done blinded except for WT in order to test the quality of transfected cells being analyzed (i.e. no cytotoxicity and normal WT-like levels)

Reporting for specific materials, systems and methods

We require information from authors about some types of materials, experimental systems and methods used in many studies. Here, indicate whether each material, system or method listed is relevant to your study. If you are not sure if a list item applies to your research, read the appropriate section before selecting a response.

Materials & experimental systems

n/a	Involved in the study	
☐	☒	Antibodies
☐	☒	Eukaryotic cell lines
☒	☐	Palaeontology and archaeology
☒	☐	Animals and other organisms
☒	☐	Human research participants
☒	☐	Clinical data
☒	☐	Dual use research of concern

Methods

n/a	Involved in the study	
☒	☐	ChIP-seq
☐	☒	Flow cytometry
☒	☐	MRI-based neuroimaging

Antibodies

Antibodies used	Myosin Light Chain 2/MLC-2v rabbit polyclonal from Proteintech Cat#10906-1-AP, Troponin T monoclonal (13-11) from Thermo Cat# MA5-12960, Goat anti-Rabbit IgG , Alexa Fluor 488 from Thermo Cat# A-11034, Goat anti-Mouse IgG, Alexa Fluor 568 Cat# A-11031, APC anti-mouse CD172a (SIRPa) from Biolegend Cat# 144013, anti-GFP-HRP (B-2) from Santa Cruz Cat# sc-9996-HRP, anti-His-HRP from Santa Cruz Cat# sc8036-HRP. Catalog numbers and dilutions provided in the methods.
Validation	All antibodies described have been previously validated and/or published including several publications by us.

Eukaryotic cell lines

Policy information about cell lines

Cell line source(s)	HEK cells were purchased from ATCC. C2C12 cells were purchased from Sigma. iPS cells (iPS-DF19-9-11T) cells were purchased from WiCell Stem Bank.
Authentication	None of the cells were authenticated by us after being received from commercial sources listed above.
Mycoplasma contamination	All cell lines tested negative for mycoplasma contamination, which was performed at the UW Biotechnology Center
Commonly misidentified lines (See ICLAC register)	*Name any commonly misidentified cell lines used in the study and provide a rationale for their use.*

Flow Cytometry

Plots

Confirm that:

☒ The axis labels state the marker and fluorochrome used (e.g. CD4-FITC).

☒ The axis scales are clearly visible. Include numbers along axes only for bottom left plot of group (a 'group' is an analysis of identical markers).

☒ All plots are contour plots with outliers or pseudocolor plots.

☒ A numerical value for number of cells or percentage (with statistics) is provided.

Methodology

Sample preparation	iPSC -dervied cardiomyocytes were generated in our lab and sample preparation for flow cytometry analysis is described in detail in the methods section.
Instrument	Attune Nxt flow cytometer
Software	FlowJo
Cell population abundance	As shown in Figure 4B, cardiomyocyte purity was ~89%. Further, cardiomyocytes were also stained with a specific marker for choosing positive cells to analyze for lamin aggregation.
Gating strategy	The gating strategy is provided in Supplementary Figure 10. Briefly, single cells were first gated using side scatter-height vs area, then gated for cardiomyocyte specific markers cTnT and MYL2v to determine population abundance.

☒ Tick this box to confirm that a figure exemplifying the gating strategy is provided in the Supplementary Information.
